# Host community activity, but not always composition, explains viral biogeography in bulk and rhizosphere soils over a tomato growing season

**DOI:** 10.1093/ismejo/wrag189

**Published:** 2026-07-15

**Authors:** Lucie Stern, Anneliek M ter Horst, Konner E Simpson-Johnson, Amélie C M Gaudin, Joanne B Emerson

**Affiliations:** Department of Plant Pathology, University of California, One Shields Avenue, Davis, CA 95616, United States; Department of Plant Pathology, University of California, One Shields Avenue, Davis, CA 95616, United States; Department of Plant Pathology, University of California, One Shields Avenue, Davis, CA 95616, United States; Department of Plant Pathology, University of California, One Shields Avenue, Davis, CA 95616, United States; Department of Plant Pathology, University of California, One Shields Avenue, Davis, CA 95616, United States

**Keywords:** Viruses, phages, viromes, soil, rhizosphere, agriculture

## Abstract

The soil microbiome is key to plant health and nutrient acquisition, and viruses likely play important but largely unknown roles in these processes. To interrogate bulk and rhizosphere soil viral biogeography, we collected samples over a tomato growing season in California from an experiment testing arbuscular mycorrhizal fungi (AMF) treatment. We generated 78 viromes, 16S rRNA gene, and ITS1 amplicon datasets, and 33 rhizosphere metatranscriptomes. Of 67 038 DNA viral “species” genomes (vOTUs), 25% were previously identified, predominantely in agricultural systems, suggesting habitat filtering and greater viral homogeneity across agricultural compared to natural soils globally. Rhizospheres had significantly higher DNA viral richness than bulk soils, whereas no significant richness differences were observed for other biota. 60% of vOTUs were shared between compartments, compared to only 21–23% of bacterial and fungal taxa. Although bulk soil viral biogeography resembled that of prokaryotes, with significant structuring by moisture content, greater virome similarity between high-moisture bulk soils and rhizospheres suggests that conditions with high host activity selected for similar viral communities. In rhizospheres, while bacterial and fungal communities differed most over time, DNA and RNA viral communities differed most by sampling location, matching prokaryotic transcriptional patterns and further implicating host activity in viral biogeography. Similarly, AMF treatment induced changes in the prokaryotic transcriptome but, across biota, only significantly affected DNA viral communities. Overall, results indicate strong viral responses to spatiotemporally localized conditions, with viral biogeography reflecting both dispersal opportunities (high between neighboring bulk and rhizosphere soils, low across fields) and selection via local host activity.

## Introduction

Soils appear to host the most diverse and dynamic viral communities globally [1], yet the ecological forces structuring these communities across soil compartments, space, time, and management remain largely understudied. Rhizospheres are hotspots of microbial activity and plant-microbe interactions, where root exudation of carbon-rich substrates shapes the activity and composition of associated microbial communities [2–5]. In return, beneficial soil microbes promote plant nutrient acquisition, disease resistance, and growth [2, 4, 6–9]. Although agriculture-associated bacteria and fungi are relatively well studied, the role of agricultural soil viruses remains comparatively overlooked, despite reviews highlighting their potential roles in microbial mortality and soil and plant health [10–14].

Compositional differences between bulk and rhizosphere soils are well established for bacterial and fungal communities but remain largely unexplored for soil viruses. Root exudation and plant-microbe interactions alter rhizosphere physiochemistry, including nutrient availability, pH, and redox conditions, in ways that strongly structure microbial communities, often selecting a distinct subset of taxa from the surrounding bulk soil [3, 15–20]. Available evidence suggests that viral communities also differ between bulk and rhizosphere soils [13, 17, 21], potentially reflecting shifts in both host composition and soil chemistry. In bulk soils, viral community composition has been linked to environmental factors such as pH, moisture, depth, altitude, and other soil chemical properties across diverse ecosystems [17, 22–27]. Whether the rhizosphere’s increased microbial activity and buffered microenvironmental conditions, particularly with respect to soil moisture dynamics [28–30], impose distinct selective pressures on viral communities, similar to those shaping bacterial and fungal hosts, remains to be investigated.

Recent advances in soil viromics, which includes purification of the viral fraction through 0.22 μm filtration and low-input DNA library construction, now enable recovery of substantially greater DNA viral diversity from soil samples [11, 26, 31], and many RNA viruses can be assessed via the conserved RdRp sequence from metatranscriptomic data [32, 33]. Together, these approaches helped to reveal that soil viral communities commonly display strong spatial and temporal structuring, particularly in natural soils (here defined as uncultivated ecosystems, such as forests, grasslands, and wetlands that, while not agriculturally managed, are often subject to low-intensity management like grazing or prescribed burns) [34]. These natural soil viral communities differ in community composition and richness over meter-scale distances, and significant differences in community structure among habitats have been demonstrated at both regional and global scales [25, 26, 35–37], with only 1.5–4% of local viral “species” (vOTUs) detected elsewhere, indicating substantial diversity and under-sampling of viruses [38, 39]. Natural soil viral communities also show higher temporal succession rates than their prokaryotic counterparts [40]. However, spatiotemporal patterns in agricultural soils remain poorly understood, with inconsistent results, derived from small sample sets (16–18 viromes) not specifically designed to measure spatiotemporal dynamics in soil viral communities [31, 41].

There is growing interest in using microbial amendments to improve soil health and crop productivity [42, 43], yet their consequences for soil microbiomes are largely unknown [44]. AMF have received considerable attention as bio-inoculants because of their widespread symbiotic associations with plants and roles in nutrient acquisition, stress tolerance, and growth promotion [45–49]. However, in intensive cropping systems, such as modern tomato production, AMF benefits are often inconsistent and strongly context-dependent, varying with soil fertility, water availability, plant genotype, and management practices [45, 50–52]. How AMF application impacts soil viral communities remains unknown, and a field-based AMF experiment provides a testable framework for assessing short-term habitat filtration impacts on viral and microbial assembly.

Despite growing interest in soil viral ecology, the ecological drivers shaping viral communities in agricultural soils and how they compare to bacterial and fungal dynamics remain poorly understood. To address this gap, the aims of this work were to: 1) investigate whether and how viral communities differ between the bulk soil and rhizosphere compartments throughout the tomato growing season; 2) determine the extent to which viral community dynamics match those of their bacterial and fungal hosts; and 3) assess how an arbuscular mycorrhizal fungal (AMF) application impacts these interactions. To address these objectives, we generated 78 viral size-fraction metagenomes (viromes) to characterize viral community composition in 36 tomato plant rhizospheres and their associated bulk soils (plus 6 bulk soils before planting) throughout one growing season in an agricultural field in Davis, CA, USA. To resolve active RNA viral communities and microbial gene expression, we sequenced 33 rhizosphere metatranscriptomes (of 36 attempted). Furthermore, we generated 78 16S rRNA gene and ITS1 amplicon sequencing datasets from the same samples to profile the bacterial and fungal host communities. To understand the effects of an arbuscular mycorrhizal fungi (AMF) treatment on these communities, the field experiment utilized a complete randomized block design, where half of the monitored tomato plants were inoculated with a commercial AMF product, while the remaining half served as controls and were inoculated with the autoclaved AMF product. Considering soil compartment, plot location, plant growth stage (time point), soil chemistry, and AMF treatment, we evaluated the extent to which viral biogeography reflected expected patterns from other microbiota, and we leveraged the previously published PIGEON v2.0 database of 443 140 vOTUs [39] as a reference framework to explore how our newly generated data fit into regional-to-global vOTU distributions.

## Materials and methods

Full methods, including detailed laboratory protocols, bioinformatic parameters, and statistical procedures, are provided in the Supplementary methods.

### Sample collection and processing

Samples were collected at the Russell Ranch Sustainable Agriculture Facility (Davis, CA, USA; 38.54’N, 121.87’W) during one tomato (Heinz 1662) growing season in 2021, where plots were arranged in a complete randomized block design. The experiment used a randomized split-plot design conducted across a subset of three 0.4 ha plots (64 × 64 m). Within each plot, the AMF-inoculated treatments and controls (inoculated with autoclaved AMF product) were assigned to the full length of 6 beds and randomized, resulting in subplots consisting of 3 beds per treatment. To strictly control for edge effects across treatment boundaries, all subsequent sample collections and measurements were conducted exclusively on the middle bed of each subplot.

Sampling was conducted at four time points: March (pre-planting; T0), June (vegetative; T1), July (flowering; T2), and August (harvest; T3). At T0, six bulk soil samples were collected (two per plot). At T1–T3, bulk and rhizosphere soils were collected from two plants per plot under each of two treatments (AMF-inoculated and control), yielding 24 samples per post-planting time point (78 total). AMF-treated seedlings were inoculated at transplanting via root-dipping into an inoculant mixture of a concentration of 10 g ha^−1^ with EndoMaxx Prime (Valent, San Ramon, CA, USA); controls received autoclaved EndoMaxx Prime. No fungicides were applied. Plots received conventional mineral fertilizer (urea ammonium nitrate; 156 kg ha^−1^) via drip irrigation throughout the season. Rhizosphere soil was defined as soil adhering to roots after excavation; bulk soil was collected 30 cm from the plant base. All samples were stored at 4°C, and laboratory processing was initiated within 48 hours of sample collection.

### Soil and plant chemistry

Bulk soil gravimetric water content was measured at each time point. Additional soil physicochemical properties (pH, organic matter, nitrate, phosphorus, potassium, sulfate, and exchangeable cations and micronutrients) were measured by Ward Laboratories (Kearney, NE, USA) using standard methods (Table S1). Plant traits assessed at harvest included vine biomass, fruit yield and quality, root AMF colonization, and foliar macro- and micronutrient concentrations. Full analytical protocols are provided in the Supplementary Methods.

### Virome DNA extraction, library construction, and shotgun sequencing

Viral fractions were extracted from 10 g of bulk soil or root-adhering rhizosphere soil resuspended in protein-supplemented phosphate-buffered saline (PPBS), as previously described [26, 31]. Briefly, soil-buffer suspensions were clarified by centrifugation and filtered through 0.22 μm polyethersulfone membranes to remove cells, and virions were pelleted by ultracentrifugation. Pellets were resuspended and treated with RNase-free DNase to remove free DNA, followed by DNA extraction using the DNeasy PowerSoil Pro kit (Qiagen). Libraries were constructed using the DNA HyperPrep kit (Kapa Biosystems-Roche) and sequenced paired-end (150 bp) on the NovaSeq S4 System (Illumina) to ~10 Gbp per virome. Full extraction and library preparation details are provided in the Supplementary Methods.

### Total DNA extraction, amplicon library construction, and sequencing

Total DNA was extracted from 0.25 g of bulk or rhizosphere soil using the DNeasy PowerSoil Pro kit (Qiagen). Amplicon libraries targeting the V4 region of the 16S rRNA gene (primers 515F/806R) and the ITS1 region (primers ITS1-F/ITS2; [53–55] were constructed following a dual-indexing strategy [56, 57] and sequenced paired-end (250 bp) on the MiSeq System (Illumina). Full PCR amplification and library preparation details are in the Supplementary Methods.

### RNA extraction, library construction, and sequencing

Each rhizosphere sample was divided into three portions at collection: one for DNA extraction (above), one flash-frozen in liquid nitrogen for RNA extraction, and one reserved for AMF colonization assessment. Total RNA was extracted from frozen rhizosphere soil using the RNeasy PowerSoil Pro Kit (Qiagen) and submitted to Genewiz (San Francisco, CA) for ribodepletion, cDNA preparation, library construction (NEBNext Ultra II RNA kit, New England Biolabs), and paired-end sequencing (150 bp) on the NovaSeq 6000 System (Illumina) to ~10 Gbp per sample.

### Bioinformatic processing

Virome reads were quality trimmed, and PhiX and host (tomato; GCA_012431665) reads removed using Trimmomatic v0.39 [58], BBMap v38.72 [59], and Bowtie2 v2.4.2 [60], respectively. Remaining reads were assembled into contigs using MEGAHIT v1.0.6 [61] with a minimum contig length of 10 kbp, and viral contigs were predicted using VIBRANT v1.2.0 [62] in virome mode. Predicted viral contigs were dereplicated into vOTUs at 95% ANI with 85% minimum alignment using dRep v3.2.0 [63]. To assess overlap with previously described vOTUs, reads were mapped to the PIGEON v2.0 database [39], and recovered PIGEON v2.0 vOTUs were clustered with study vOTUs using the same dereplication settings. Reads were mapped to the final non-redundant vOTU set using Bowtie2, and a coverage table was generated using CoverM v0.6.1 [64], with mean coverage and a minimum breadth of 75%. The resulting non-redundant dataset of vOTUs was taxonomically annotated using geNomad v1.11.1 [65].

Paired-end amplicon reads were assembled and denoised into ASVs using PANDAseq v2.9 [66] and DADA2 v1.12.1 [67]. Taxonomy was assigned using the RDP classifier with the SILVA v132 database [68] for 16S rRNA gene sequences and the UNITE v2021-05-10 database [69] ITS sequences. Sequences matching mitochondria or chloroplasts were removed and ASV tables converted to relative abundances prior to downstream analyses.

Metatranscriptomic reads were trimmed and depleted of PhiX and host reads as described for viromes. For RNA viral community analyses, remaining reads were co-assembled by plot location and treatment (AMF-amended vs. AMF control), yielding 6 co-assemblies, using MEGAHIT. Proteins were predicted with Prodigal v2.6.3 [70] and RdRp sequences recovered using HMMER v3.3.2 [71] as previously described [33, 72]. Predicted proteins were further annotated using DIAMOND v0.9.22.123 [73] against the NCBI nr prokaryotic database (v203), using a E-value threshold of 1e-06. Contigs containing prokaryotic genes were flagged and reads mapping to these contigs were removed to reduce prokaryotic RNA signal. The remaining reads were co-assembled again using MEGAHIT, and this process was iterated three times. The final set of contigs containing predicted RdRp sequences was clustered using dRep [63] at 95% ANI with a minimum alignment of 85%, using the ANImf algorithm. Metatranscriptomic reads were mapped back to these sequences and to RefSeq v203 RNA viral sequences (n = 4472) using Bowtie2, and a coverage table was generated using CoverM [64] with the same parameters as described for DNA viromes. For community transcriptome analyses, rRNA reads were removed using SortMeRNA v4.3.7 [74]. Contigs generated from the plot location and treatment condition co-assemblies described above were concatenated into a single unified reference transcriptome database. Non-rRNA reads were mapped back to this reference database, and transcript abundance was quantified using Salmon v1.10.3 [75]. Functional annotation was performed using eggNOG-mapper v2.1.13 [76]. All bioinformatic scripts are available at https://github.com/AnneliektH/TomatoRhizo and https://github.com/ljiraska/Tomato_rhizo_AMF.

### Statistical analysis

All statistical analyses were performed in R v4.5.2 [77] using phyloseq v1.52.0 [78], vegan v2.7.2 [79], and ggplot2 v4.0.3 [80]. ASV tables were CSS normalized and all abundance tables Hellinger transformed prior to analysis. Statistical significance was defined as p < 0.05 for single comparisons and adjusted p < 0.05 (Benjamini-Hochberg correction) for multiple comparisons. Alpha diversity (observed richness) was compared between compartments using Wilcoxon rank-sum tests. Beta diversity was assessed using principal coordinate analysis (PCoA) with Bray–Curtis dissimilarity. Permutational multivariate analysis of variance (PERMANOVA; adonis2, 999 permutations) tested effects of compartment, time point, plot location, and AMF treatment on community composition, with two- and three-way interactions tested where appropriate. Mantel tests assessed correlations between community and soil chemistry dissimilarity matrices (999 permutations, Pearson correlation). Linear regressions and Pearson correlations assessed relationships between PCoA axes and environmental variables. Variation partitioning (varpart, vegan) decomposed community variation among soil moisture, soil chemistry, location, and time, with forward selection of soil chemistry variables using adespatial [81]. Temporal effects were assessed using both categorical time points and linear time. Differential gene expression analysis was performed using DESeq2 v1.48.2 [82] with a design accounting for site, time point, and treatment; differentially expressed genes were identified at adjusted *P* value <0.05 and |log2FC| > 1. Desiccation stress responses were evaluated using 22 previously identified desiccation-upregulated genes [83], with temporal trends modeled using linear mixed-effects models (lme4; [84]. All data processing and statistical analysis scripts are available on Github (https://github.com/ljiraska/Tomato_rhizo_AMF). Full statistical details are provided in the Supplementary Methods.

## Results and discussion

### Dataset overview

A total of 67 038 viral operational taxonomic units (vOTUs, approximately species-level taxonomy; [85]) was recovered from the 78 viromes, of which the vast majority (98%) were taxonomically assigned to the tailed bacteriophage class *Caudoviricetes* (Table S2) via geNomad [65]. We recovered 380 viral RNA-dependent RNA polymerase (RdRp) genes [33, 72, 32] from the 33 rhizosphere metatranscriptomes. Based on the taxonomic classification of these RdRp sequences against profile hidden Markov models (HMMs) of known viral families (see Methods), the dataset was dominated by predicted viruses of fungi and prokaryotes. Specifically, there were 174 putative viruses of fungi, 166 of prokaryotes, 20 of insects and/or vertebrates, 15 of other animals and/or plants, and only 3 confidently assigned to plants alone. Of 1 051 814 total transcripts recovered, ~30% were functionally annotated, of which the vast majority were bacterial in origin (96%); downstream analyses therefore interpret community-wide gene expression as reflective of bacterial transcription. A total of 16 146 16S rRNA gene and 6684 ITS1 amplicon sequence variants (ASVs) were obtained from the same 78 samples as the DNA viromes.

### Agricultural soil viral “species” (vOTUs) show more homogeneous detection across similar habitats than do those from natural soils

To investigate the global habitat distribution of vOTUs recovered at our field site (Russell Ranch), we leveraged the PIGEON v2.0 database [39] of 443 140 vOTUs from a variety of ecosystems (Fig. 1). Of the 67 038 vOTUs recovered here, 16 452 (25%) were from PIGEON v2.0 and thus previously detected in other studies, including 10 173 (15%) from the same field site in 2018 (vs. 2021 here), consistent with prior observations of temporal stability at this site [41] and suggesting that viral populations can be detected over long periods in agricultural systems. Although we cannot definitively distinguish whether the recurrence of the same vOTUs in 2018 and 2021 was due to passive virion persistence (sampling from the same pool of viral particles) or the active maintenance of viral populations via repeated host infection cycles, recent studies suggest that ongoing infections were more likely. For example, microcosm work by DiPietro et al. [86] demonstrated that phage decay rates can range from 0.11% to 2.07% per hour in autoclaved soils, with a relatively rapid loss of infectivity over 2–37 days in the absence of a host. Additionally, microcosm studies of grassland soil viral communities indicate near-complete viral community turnover (85% distinct vOTUs) over 10 days [40], suggesting that most viral particles did not persist over that time period. Together with prior observations of temporal stability of vOTUs at the same agricultural field site [41], these results suggest that viral populations here were likely actively maintained through punctuated and/or ongoing host infection cycles, rather than via passive persistence as virions over ~3 years.

**Figure 1:**
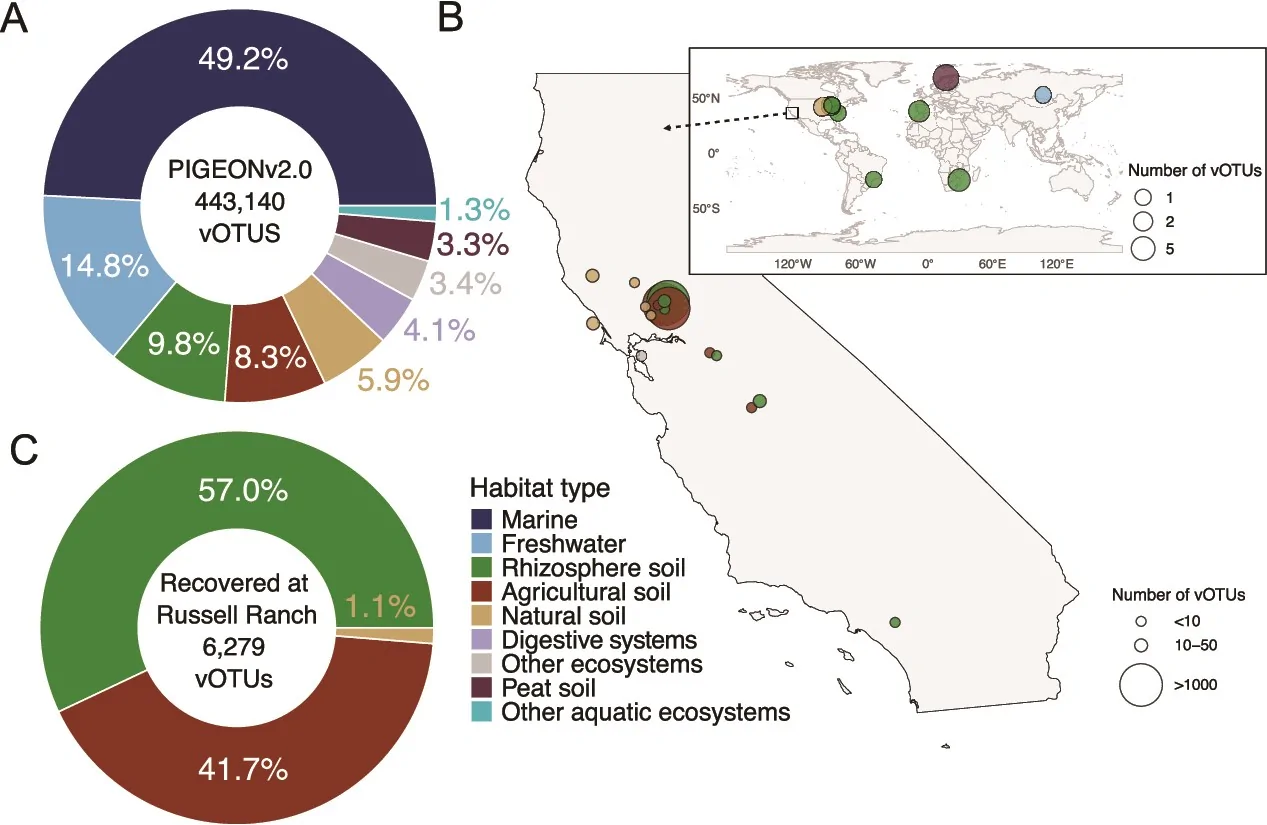
Global and California-wide distribution of Russell Ranch vOTUs, recovered using the PIGEON v2.0 database. A: Composition of the PIGEON v2.0 database of 443 140 vOTU sequences, colored by environment, excluding vOTUs from Russell Ranch. B: Global and California-wide locations of vOTUs (n = 6279) recovered at Russell Ranch by read mapping to PIGEON v2.0 based on where they were first identified. C: Relative proportions of vOTUs (n = 6279) recovered at Russell Ranch by read mapping to PIGEON v2.0, grouped and colored by the environment from which each vOTU was first recovered; ~0.2% of vOTUs are not depicted in C (slices too small to visualize: 0.1% peat, 0.02% freshwater, 0.06% other ecosystems) but appear in B.

Excluding vOTUs previously detected at Russell Ranch, the remaining 6279 vOTUs (11%) from PIGEON v2.0 were predominantly recovered from other agricultural sites, in both bulk and rhizosphere soil (Fig. 1B and 1C). Of those, 98% were from other agricultural systems in California, mostly from almond orchard soils (77%; [87]), though this likely reflects geographic sampling bias, given that 90% of soil vOTUs in PIGEON v2.0 are from California, and soil viromes have not been extensively reported from other locations. Still, vOTU recovery predominantly from agricultural systems, which represent only 18% of PIGEON v2.0, suggests habitat-specific adaptation. Consistent with strong habitat boundaries, no vOTUs from marine ecosystems were recovered here and only one from freshwater, contrasting with wetland viromes where 46% of previously detected vOTUs were from aquatic environments [39].

Among vOTUs originally described from rhizosphere studies, most (53%) were detected in both rhizosphere and bulk soils here, and most (64%) of the vOTUs previously detected from agricultural soils were shared between compartments, suggesting that many agricultural soil viruses are compartment generalists. These patterns are consistent with agriculture-specific habitat filtering, extending prior evidence of habitat specificity in peat, wetlands, and burned soils [26, 38, 39] to agricultural systems.

Though more sampling is needed for broad generalizability, 15% of vOTUs here were previously detected elsewhere (excluding the same field site), compared to only 1.5–4% in prior studies of natural systems [26, 39, 88]. Although sampling bias is clearly a contributing factor, these results suggest that agricultural soil viral “species” have comparatively more homogeneous regional-to-global distributions than those in natural soils, consistent with stronger habitat filtering in managed systems.

### Viral communities in bulk and rhizosphere soils diverged over the course of the tomato growing season

To interrogate the main drivers of community structure in our dataset, we first explored which variables shaped the viral, bacterial, and fungal communities overall, across all time points, locations, compartments (bulk vs. rhizosphere), and AMF treatment. Overarching patterns were similar across biota, with time, compartment, and plot (location) all contributing factors explaining DNA viral, bacterial, and fungal community composition, followed by marginal effects of AMF treatment and variable interactions (Tables S1–S4). However, the proportions of variance explained (*R*^2^ values) were much higher for viruses (*R*^2^ = 0.16, 0.15, 0.14 for time, compartment, and location, respectively), with 80% of the total variation in DNA viral communities explained by these factors or their combination (Fig. 2A and Table S3), compared to 60% for bacterial and fungal communities, where no single factor exceeded *R*^2^ = 0.12 (Fig. S1, Table S3). Mantel tests revealed a strong correlation between bacterial and fungal community beta-diversity (*r* = 0.67, *P* value = 0.001) and a significant but weaker correlation between DNA viral and bacterial beta-diversity (*r* = 0.33, *P* value = 0.001; Table S5). Together, these results indicate that viral communities more strongly reflected spatiotemporally localized conditions than did bacterial or fungal communities, and that host community composition alone did not fully explain viral biogeography.

**Figure 2:**
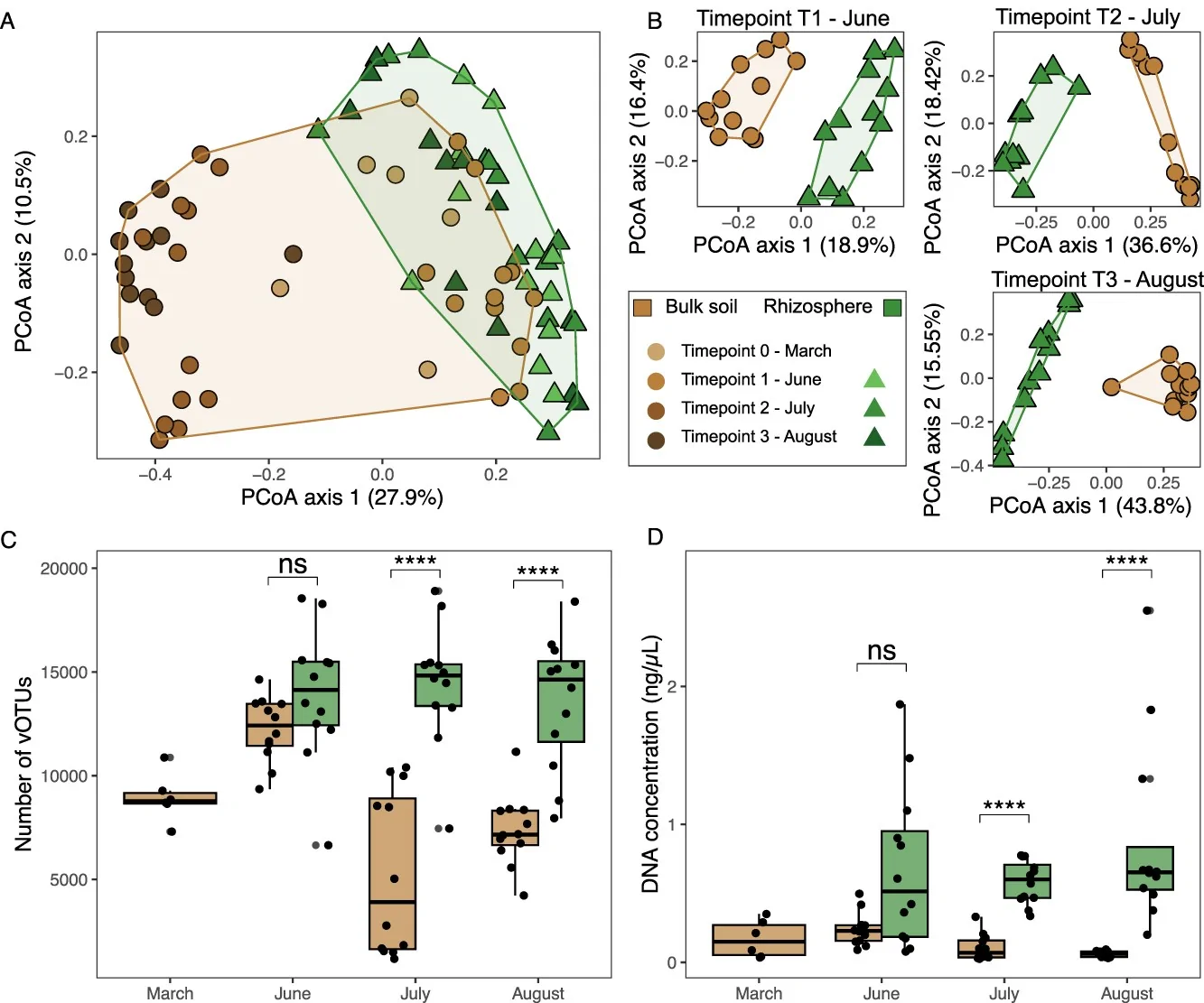
Bulk and rhizosphere soil viral community composition, viral “species” (vOTU) richness, and viromic DNA concentrations. (A) Principal coordinates analysis (PCoA) of DNA viral communities across 78 viromes, based on Bray–Curtis dissimilarities of vOTU relative abundances. Points are colored by soil compartment (bulk soil vs. rhizosphere) with shades representing sampling time points: T0 (March, pre-planting), T1 (June, 6 weeks after planting), T2 (July, 10 weeks after planting), and T3 (August, 17 weeks after planting), according to the panel B legend. Axis labels indicate the percentage of total variance explained. (B) Separate PCoAs of DNA viral communities, faceted by sampling time point and colored by soil compartment. (C, D) Boxplots of (C) total vOTU richness (number of vOTUs) per virome per time point in bulk and rhizosphere soils and (D) DNA concentrations (a proxy for viral particle abundances) obtained from virome extractions from bulk and rhizosphere soils across sampling time points, colored by soil compartment, as in (B). Boxes represent the interquartile range (IQR, 25th–75th percentile), the central line shows the median, whiskers extend to 1.5× IQR, and individual points outside whiskers are outliers. Points are jittered for clarity. Statistical significance between bulk and rhizosphere vOTU richness (C) and DNA concentration (D) was assessed using a Wilcoxon rank-sum test and is indicated where applicable: n.s., not significant; ^*^P < 0.05; ^**^P < 0.01; ^***^P < 0.001; ^****^P < 0.0001.

Although DNA viral community composition differed significantly between bulk and rhizosphere soils, the strongest compositional separation was between later season bulk soils (July and August) and all other viromes (March and June bulk soils and all rhizospheres together; Figures 2AB). Viral communities showed greater compartmental overlap early in the season, with increasing divergence later in the growing season, a pattern consistent with but less pronounced in bacterial and fungal communities (Fig. 2B and S1CD). Overall, 60% of vOTUs were shared between compartments, compared to only 21% and 23% of bacterial and fungal ASVs, respectively (Fig. S2). There were no significant differences in richness between compartments for bacteria or fungi (Fig. S1EF), but viral richness was significantly higher in rhizospheres at the last two time points, with bulk soil richness decreasing over time while rhizosphere richness remained stable and high (Fig. 2C), a pattern mirrored by viromic DNA yields, an established proxy for viral biomass (Fig. 2D; [40]). Our results are consistent with strong compartmental differentiation reported from viral sequences mined from total metagenomes of late-season maize rhizosphere and bulk soils [17], but suggest these differences may be less pronounced earlier in the growing season. Overall, despite more shared vOTUs between compartments and higher rhizosphere viral richness, the broad biogeographical patterns for DNA viruses resembled those of their likely host prokaryotes.

### Bulk soil viral communities responded significantly to changes in soil moisture, likely indirectly via host community responses and activity

We next sought to explore the relative importance of time, plot location, and AMF treatment (key experimental design factors that could be reflective of succession, dispersal limitation, and environmental selection, respectively) in structuring bulk soil viral communities. These were primarily structured by time point (*R*^2^ = 0.41, *P* value = 0.001; Fig. 3A and 3B) and secondarily by plot location (*R*^2^ = 0.15, *P* value = 0.001; Fig. S3A), followed by a weaker effect of AMF treatment (*R*^2^ = 0.03, *P* value = 0.007; Fig. S3B), as well as interactions among variables (Table S6). Bulk soil bacterial and fungal communities showed similar patterns, with no significant effect of AMF treatment (Table S6, Fig. S3C–F). This suggests that, when evaluated across broad experimental design factors (i.e., time point and plot location), bulk soil viral community dynamics were similar to those of their likely host bacteria (Table S4).

**Figure 3:**
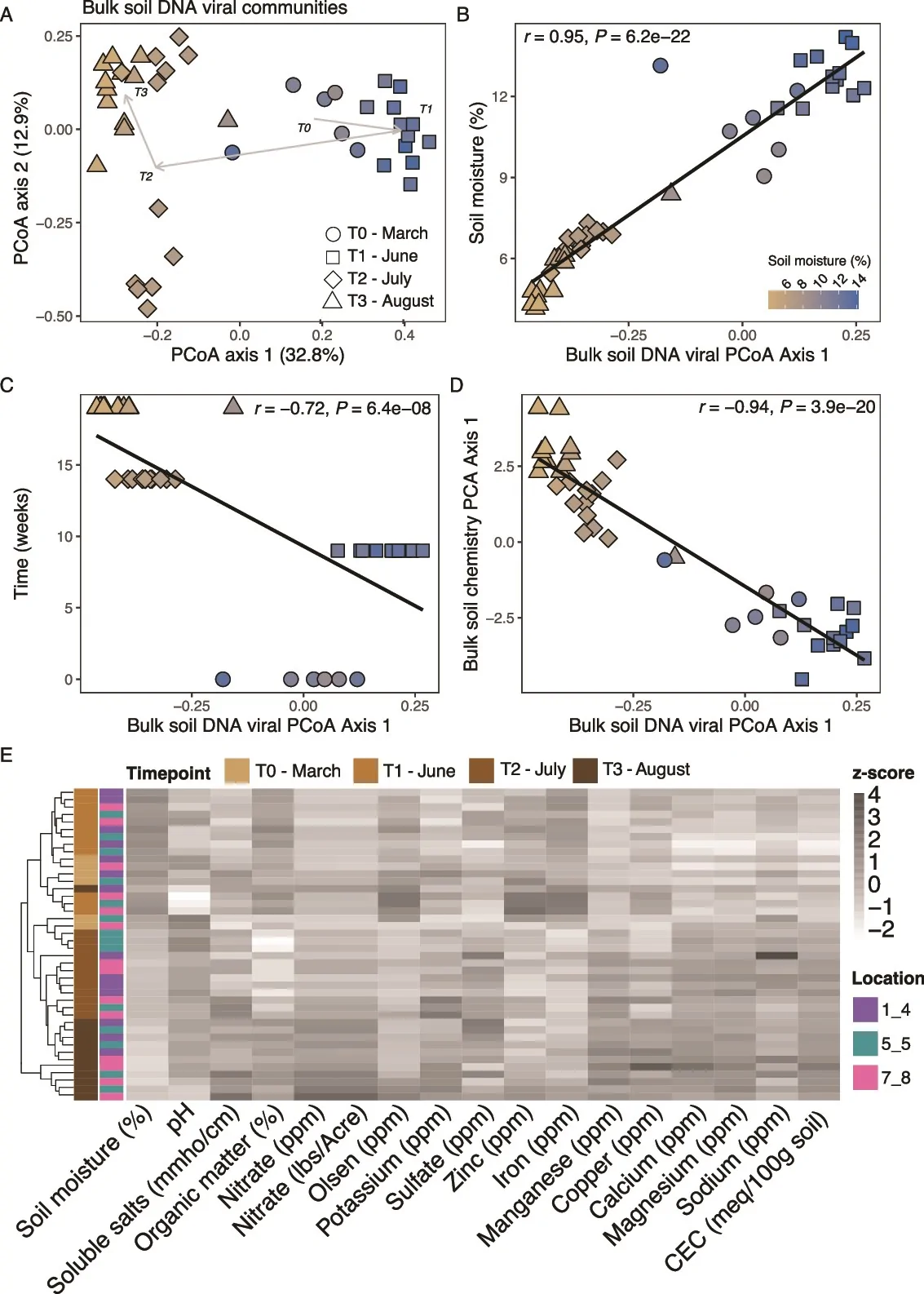
Bulk soil DNA viral community composition over time, moisture content, and other soil physicochemical properties. (A) Principal coordinates analysis (PCoA) of DNA viral community composition, based on Bray-Curtis dissimilarities of vOTU relative abundances, colored by bulk soil moisture content (according to the legend in B), with shapes indicating time point. (B-D) Linear regression analyses, comparing PCoA axis 1 of panel A (a proxy for bulk soil DNA viral community composition) to (B) soil moisture content, (C) time of sample collection, and (D) a proxy for overall physicochemistry: the first axis of a principal component analysis (PCA axis 1, plot in Fig. S3G) of all bulk soil physicochemistry measurements (shown in E), excluding soil moisture, with correlation statistics for (B-D) inset in each graph. (E) Hierarchical clustering of samples, based on z-scores of measured bulk soil physicochemical variables. mmho/cm, millimhos per centimeter; ppm, parts per million; CEC, cation exchange capacity; meq/100 g, milliequivalents per 100 grams of soil.

The compositional differences among time points raised the question of whether temporal changes reflected a directional successional trajectory of community turnover or instead consisted of discrete, non-directional shifts, potentially driven by selection imposed by other variables, such as changing physicochemical conditions. We thus tested whether treating time as a linear variable (weeks since the start of sampling) versus as discrete categorical sampling time points better explained community composition (Table S7). Categorical time points explained approximately twice as much variation in the DNA viral communities as linear time (with bacterial and fungal communities showing similar patterns), suggesting that the influence of time point reflected fluctuating environmental conditions and associated selective pressures (by way of hosts), rather than iterative temporal turnover.

The observed differences in bulk soil viral communities across time points, including divergence from rhizospheres at the last two time points, coincided with declining soil moisture late in the growing season, dropping from an average of 12% in March and June to 6% in July and August. PCoA axis 1 of bulk soil viral communities was strongly correlated with soil moisture (*r* = 0.95, *P* value <0.01; Fig. 3A-B) and less strongly with linear time (*r* = −0.72, *P* value <0.001; Fig. 3C), with comparable moisture correlations for bacterial (*r* = 0.84, *P* value <0.001; Fig. S4A) and fungal communities (*r* = 0.79, *P* value <0.001; Fig. S4B), likely indicating an indirect viral response to soil moisture by way of more direct host responses. However, while viral richness (*r* = 0.43, *P* value <0.001; Fig. S4C) and viromic DNA yields (*r* = 0.34, *P* value <0.001; Fig. S4D) were also positively correlated with soil moisture, no comparable relationship emerged for bacterial or fungal richness (Fig. S4E-F). Together, these patterns suggest a strong dependence of DNA viruses and their presumed hosts on soil moisture, consistent with previous studies showing that moisture regulates viral abundance, persistence, and mobilization in soils [14, 27, 37, 40, 89].

Although soil moisture emerged as a clear explanatory factor for bulk soil viruses, the relative contributions of host community composition and other soil chemistry variables required further investigation. All microbial groups showed significant correlations with one another, with the strongest associations between bacterial and fungal communities (Mantel *r* = 0.72, *P* value = 0.001) and, for DNA viruses, with bacterial communities (Mantel *r* = 0.57, *P* value = 0.001; Table S5). In bulk soils, viral communities showed stronger correlations with soil physicochemical properties (Mantel *r* = 0.59, *P* value = 0.001; Fig. 3D) than bacterial (*r* = 0.33, *P* value = 0.001) or fungal communities (*P* value = 0.138; Table S5), and soil chemistry without moisture explained viral composition almost as strongly (*r* = −0.94, *P* value <0.001; Fig. 3D) as moisture alone (*r* = −0.93, *P* value <0.001). One T3 sample grouped with earlier time points across all ordinations, driven by soil chemistry that was divergent from other samples from the same time point (Fig. 3E).

Variance partitioning analysis, treating soil moisture separately from other soil chemical variables, retained only pH and nitrate as significant predictors of DNA viral communities (Table S8), consistent with previous work showing that pH and soil chemistry can influence viral attachment, persistence, and virus-host encounter and infection rates in soils [14, 90–92]. Variance partitioning also revealed strong covariance among time point, soil moisture, and soil chemistry, explaining why categorical time points, but not linear time, captured most of the variation in bulk soil DNA viral communities.

Together, these results may explain why bulk soil viral communities most closely resembled rhizospheres early in the growing season, when soil moisture was high: both high-moisture bulk soils and rhizospheres are known hotspots of microbial activity [16, 91–97]. We hypothesize that higher early-season moisture supported greater host activity and viral mobility (dispersal) between compartments, resulting in more homogeneous viral communities. Soil moisture is already a well-established driver of soil viral community structure [27, 40] and of microbial activity more broadly [93–95]. Rhizosphere soils are known to retain water during drying periods while bulk soils experience greater moisture fluctuations [28, 30]. Consistent with sustained rhizosphere moisture throughout the season, metatranscriptome data from control rhizosphere samples that were inoculated with autoclaved AMF showed no evidence of increasing desiccation stress response by increasing transcription in hallmark bacterial desiccation genes over time (*P* value >0.05, Table S9; [83], indicating that rhizosphere microbiomes were not experiencing water limitation. This supports the concept of the rhizosphere as a buffered microenvironment where plant-derived water and carbon inputs maintain microbial activity even as bulk soil conditions fluctuate. Microbial activity has been hypothesized to be an important driver of soil viral community composition [25, 40], and here we suggest that microbial activity could be a stronger selective force in viral community assembly than the habitat-specific selection imposed by soil compartment.

### Spatial structuring was the dominant feature of rhizosphere DNA and RNA viral communities, reflecting prokaryotic activity patterns more than community composition

We next examined the ecological drivers of rhizosphere viral community structure to assess whether patterns observed in bulk soils also extend to the plant-root interface. Unlike bulk soils, which were dominated by time point (and associated moisture and chemistry differences), rhizosphere DNA viral communities were primarily structured by plot location (*R*^2^ = 0.27, *P* value = 0.001; Fig. 4A), followed by AMF treatment (*R*^2^ = 0.14, *P* value = 0.001; Fig. 4B), with a smaller but significant effect of sampling time point (*R*^2^ = 0.08, *P* value = 0.003; Table S10). Rhizosphere RNA viral communities showed similar spatial structuring, also differing significantly by plot location (*R*^2^ = 0.10, *P* value = 0.001), time point (*R*^2^ = 0.08, *P* value = 0.005), and their interaction (*R*^2^ = 0.13, *P* value = 0.034; Table S10). These compositional differences among locations at the same field site are consistent with previous reports of strong spatial structuring of soil viral communities at local scales [25, 35, 98] and suggest dispersal limitation as an important factor in rhizosphere viral community assembly.

**Figure 4:**
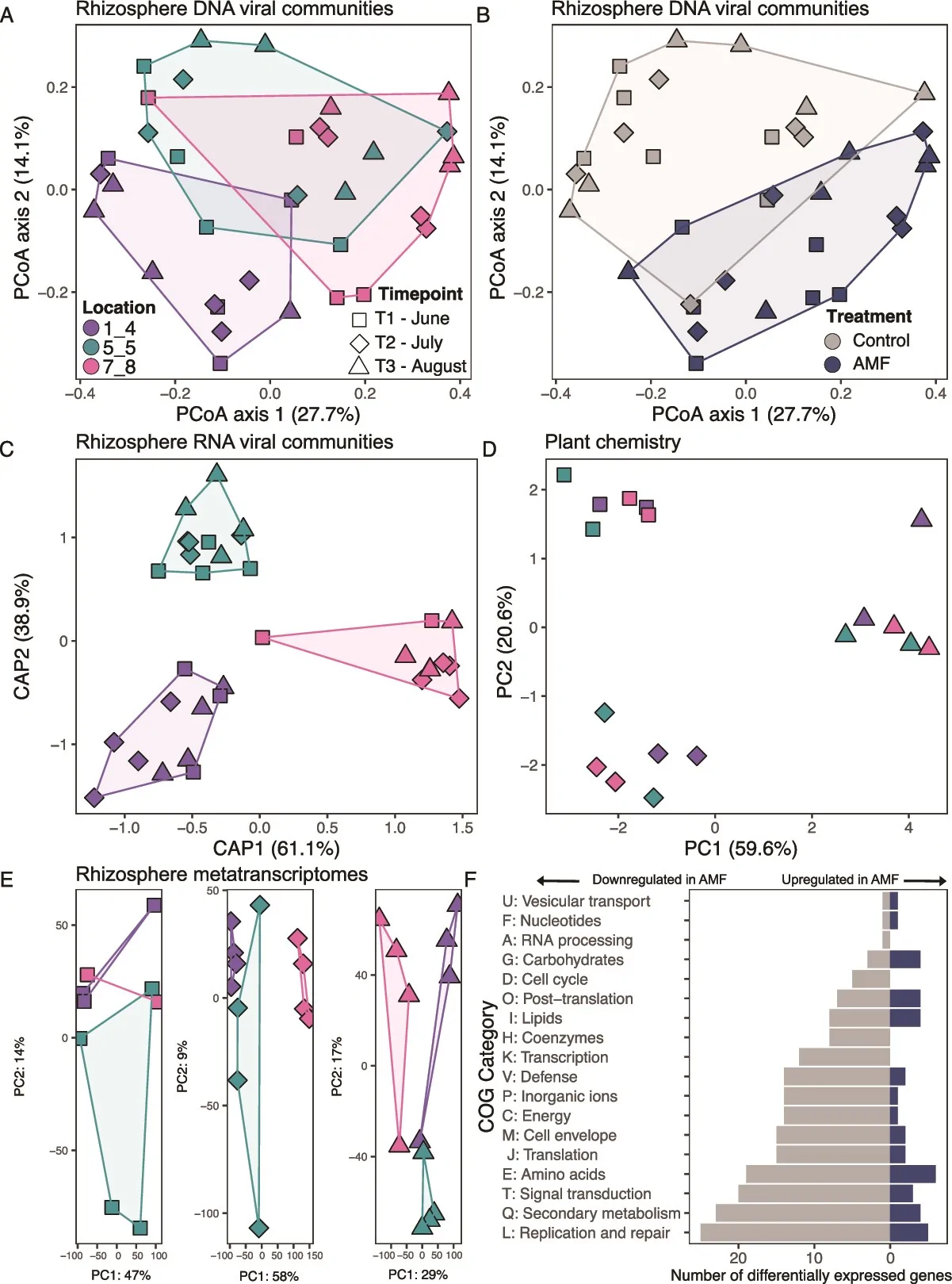
Rhizosphere DNA and RNA viral biogeography and explanatory factors. (A,B) Principal coordinates analysis (PCoA) of rhizosphere DNA viral community composition, based on Bray-Curtis dissimilarities of vOTU relative abundances (same plot in A and B, colored by plot location in A) and AMF treatment condition in B), (C) constrained analysis of principal coordinates (CAP) of rhizosphere RNA viral communities, based on Bray-Curtis dissimilarities of RNA-dependent RNA polymerase (RdRP) gene relative abundances, (D) principal components analysis (PCA) of a suite of 13 z-score-standardized plant aboveground chemistry measurements (Table S11), (E) PCA of rhizosphere metatranscriptomic data, based on read mapping to individual genes assembled from the metatranscriptomes (predominantly prokaryotic data, as >96% of the genes were predicted to be derived from prokaryotes). Each gene was treated as a separate feature, and gene relative abundances across samples were used to calculate Euclidean distances. (F) Number of differentially expressed prokaryotic genes between AMF treatment and control samples, grouped by COG category (full category descriptions provided in Table S12). Shapes in all ordination graphs (A-E) correspond to time point. Colors in A, C, D, and E correspond to plot location, according to the legend in A). Colors in B and F correspond to AMF treatment, according to the legend in B). For PCA and PCoA, percentage values indicate the proportion of total variance in the dataset explained by each axis. For CAP, axes are constrained to maximize separation between predefined groups, and percentages sum to 100% of the variance that can be attributed to group differences, not total variance in the dataset.

In contrast to the viral communities, rhizosphere bacterial and fungal communities were structured primarily by time point, likely reflecting shifts in plant growth stage and root exudate composition [99–101], consistent with significant differences in plant chemistry over time but not location (Fig. 4D, Tables S10 and S11). While compositional similarities within the same field were unsurprising given that rhizosphere bacterial communities from the same plant species tend to be relatively uniform across broad geographic regions [19, 102], the decoupled biogeographical patterns between viral and host communities represent a new observation in the rhizosphere. Despite these differing biogeographical patterns, Mantel tests confirmed that rhizosphere DNA viral communities still correlated strongly with both prokaryotic and fungal communities (*r* = 0.35–0.37, *P* value <0.001), though prokaryotes and fungi were more strongly correlated with each other (*r* = 0.56, *P* value <0.001; Table S5). RNA viral communities showed no significant correlations with prokaryotes or fungi, even in subgroup analyses by predicted host taxonomy (Table S5), likely reflecting the broad host range of RNA viruses across all domains of life. More consistent with the viral community spatial patterns, metatranscriptomic data (derived from transcript mapping to assembled metatranscriptomic data) were most strongly differentiated by plot location, followed by time point and AMF treatment (Fig. 4E, Table S10), with divergence among sampling locations increasing later in the season. As the transcripts predominantly reflected prokaryotic activity, these results are consistent with host activity as a key driver of rhizosphere viral community composition, particularly for DNA viruses.

Overall, we suggest that the observed rhizosphere viral biogeographical patterns likely arose from multiple features of viruses and their biotic and abiotic requirements that may differ from those of their hosts. Spatial structuring indicates that dispersal limitation had a strong impact on rhizosphere viral community assembly. Unlike their bacterial hosts, which can actively migrate or track root exudates [103], extracellular virions rely on passive dispersal that can be restricted by porewater transport pathways and electrostatic adsorption onto soil clay minerals and organic matter surfaces [12, 104, 105]. This physical immobility is further compounded by short viral particle residence times (estimated to be on the scale of days, based on five model phages in autoclaved soil microcosms [40, 86]), resulting in spatial structuring observed in both natural and agricultural soils [25, 31]. For a productive infection, a viral particle needs to first encounter (by way of passive dispersal) a suitable host cell, and then that host cell needs to be sufficiently physiologically active for an infection to proceed [14]. Temperate phages (those capable of lysogeny) similarly rely on host cell activity for lysis-lysogeny decisions [14], such that both lytic and temperate phage responses to host activity could be captured in the viral particle fraction (as measured in viromes here) and reflected in biogeographical patterns. Altogether, these additional limitations and selective pressures on viruses resulted in decoupled biogeography between rhizosphere DNA viruses and their host prokaryotes, with spatial structuring and host activity patterns best predicting rhizosphere viral community composition.

### AMF treatment induced changes in bacterial transcription that correlated with changes in the rhizosphere DNA viral community

Although AMF application did not significantly affect root colonization rates or measured plant traits at harvest (Fig. S5, Table S11), it did have a significant effect on rhizosphere DNA viral community composition (R^2^ = 0.14, *P* value = 0.001; Fig. 4B; Table S10) but only a marginal effect in bulk soils (Table S6). In contrast, AMF treatment had no detectable effect on bulk soil bacterial or fungal community composition and only a marginal effect on those communities in the rhizosphere (Tables S6 and S10), consistent with findings from other soil systems where AMF alone exerted limited influence on microbial communities unless combined with organic amendments [106]. However, metatranscriptomic analyses revealed a clear transcriptional response to AMF in the rhizosphere (Tables S10 and S12), with differential expression analysis (*P*_adj_ value <0.05, |log2FC| > 1; n = 496 genes) showing that transcripts across multiple functional categories (replication and repair, secondary metabolism, signal transduction) were broadly downregulated under AMF treatment (Fig. 4F, Table S12), though ~40% lacked functional annotation. Twenty-two desiccation and stress-related genes showed significantly increasing expression over time in AMF-treated plants but not in controls (Table S9), resembling the well-documented priming effect in AMF-colonized plants, where stress-responsive genes are upregulated under non-stress conditions to enable faster subsequent responses [107–109]. Our results suggest that a similar priming phenomenon may occur in the rhizosphere and indicate that AMF treatment influenced bacterial host physiology and activity more than community structure. As these functional changes co-occurred with shifts in DNA viral community composition, they provide additional evidence that DNA viral community composition was closely tied to host metabolic state and activity, rather than to host taxonomic composition alone.

## Conclusions

Here, we investigated viral community assembly and its ecological drivers in tomato bulk and rhizosphere soils over one growing season in California, USA, revealing strong ties between viral biogeography, measured soil properties, and prokaryotic activity patterns. Agricultural soil viruses showed strict habitat boundaries, whereby nearly all previously known vOTUs were originally detected in other agricultural rather than natural ecosystems, implicating habitat filtering as a mechanism for maintaining stable or recurrent viral populations across agricultural ecosystems.

Viral communities were governed by distinct ecological processes compared to their microbial hosts. Expected differences between bulk and rhizosphere soils emerged for all measured microbial communities (prokaryotes, fungi, and viruses), but compositional separation between compartments increased over the growing season, most strongly for viruses. Bacterial and fungal communities responded primarily to temporal dynamics in both compartments, likely reflecting seasonal shifts in soil chemistry and plant phenology. In contrast, viral community assembly drivers were compartment-specific: bulk soil viral communities tracked temporal changes in soil moisture and chemistry, while rhizosphere viruses were structured by spatial location and AMF treatment. This decoupling between virus and host biogeographical patterns suggests that viruses experience stronger spatial constraints than do their larger hosts, challenging conventional assumptions about size-related dispersal in soils [25, 110]. AMF treatment further showed decoupled patterns between virus and host communities, with significant restructuring of rhizosphere viral but not bacterial or fungal composition. However, AMF treatment induced widespread transcriptional shifts in the rhizosphere microbiome, including stress-gene priming, corresponding to previously documented plant responses to mycorrhizal colonization, suggesting that viruses track host metabolic state rather than taxonomic composition, responding to functional changes that remain invisible in standard community profiling. Together, these findings position soil viruses as dynamically responsive yet spatially constrained components of the soil microbiome, governed by ecological rules distinct from those shaping bacterial and fungal communities, and they underscore the need to integrate virus-specific ecology into broader frameworks for understanding soil microbial dynamics.

## Supplementary Material

Supplementary_material_wrag189

## Data Availability

Raw sequencing reads for viromes, metatranscriptomes, 16S rRNA gene, ITS amplicon libraries, are available on NCBI under BioProject PRJNA937255. Final set of vOTU sequences and RdRps are available on Dryad (https://datadryad.org/), using the following link https://doi.org/10.5061/dryad.sf7m0cgnb. vOTU sequences are also available on Dryad within the PIGEON v2.0 database (https://doi.org/10.25338/B8C934).
