# Supplementary material for: Host community activity, but not always composition, explains viral biogeography in bulk and rhizosphere soils over a tomato growing season
Source: ISME J. 2026 Jul 15;20(1):wrag189. doi: 10.1093/ismejo/wrag189 (PMC13671227; doi:10.1093/ismejo/wrag189)
Supplement: Supplementary file 1 [file supplementary_material_wrag189.zip › Supplementary_materials_and_figures_06_24_2026.pdf]

Title:

Host community activity, but not always composition, explains viral biogeography in bulk and rhizosphere soils over a tomato growing season

Authors:

Lucie Stern[1] & Anneliek M. ter Horst[1] *, Konner E. Simpson-Johnson[1], Amélie C.M. Gaudin[1], Joanne B. Emerson[1]

* Co-first authors

[1] Department of Plant Pathology, University of California, One Shields Avenue, Davis, CA, 95616, USA

Supplementary materials and methods

Sample collection and processing

Samples were collected at the Russell Ranch Sustainable Agriculture Facility (Davis, CA, USA; 38.54'N, 121.87'W) during one tomato (Heinz 1662) growing season in 2021, where plots are arranged in complete randomized block design. The experiment used a randomized split-plot design conducted across a subset of three 0.4 ha plots (64×64 m). Within each plot, the AMF-inoculated treatments and controls (inoculated with autoclaved AMF product) were assigned to the full length of 6 beds and randomized, resulting in subplots consisting of 3 beds per treatment. To strictly control for edge effects across treatment boundaries, all subsequent sample collections and measurements were conducted exclusively on the middle bed of each subplot. Sampling was conducted at four time points: March (pre-planting; T0; 0 weeks), June (vegetative phase, 6 weeks post-planting; T1; 9 weeks since T0), July (Flowering, 10 weeks post-planting; T2; 14 weeks since T0), and August (maturity, harvest, 16 weeks post-planting; T3; 19 weeks since T0). In total, 78 soil samples were collected. At the pre-planting time point (March), six bulk soil samples were collected (two per plot). At each post-planting time point (June, July, and August), 24 samples were collected, comprising bulk and rhizosphere soils from two plants per plot under two treatment conditions (with and without arbuscular mycorrhizal fungi, AMF). Across post-planting time points, this resulted in sampling of three plots × two soil compartments (bulk and

rhizosphere) × two plants per treatment × two treatments. Each plot was conventionally managed and left fallow during the preceding winter months, then received mineral fertilizer (urea ammonium nitrate solution; 156 kg ha^{-1} total) via drip line irrigation 3-4 times throughout the growing season. Tomato seedlings with the first two true leaves were transplanted into the field. All seedlings assigned to the AMF treatment were inoculated using a root-dip method with EndoMaxx Prime (Valent, San Ramon, CA, USA) at transplanting, following the manufacturer's instructions. Control seedlings were dip-inoculated with autoclaved EndoMaxx Prime. Fertilization and field management were identical across treatments. No soil biocides (ie. fungicides) were applied to allow AMF establishment and soil microbiome characterization; no disease pressure was observed. At each post-planting sampling time point (June, July, August), two plants per plot were randomly selected for each treatment (with and without AMF), and their rhizosphere (soil adhering to roots after excavation) and accompanying bulk soils (30 cm from the base of the plant) were collected and processed according to the descriptions below. At the pre-planting time point (March), no plants were present and only bulk soil was sampled, two per plot, in approximately the same location (i.e., 30 cm from where plants were to be planted). Bulk soil samples were collected using a soil knife from the upper approx. 25 cm of soil (2.5 × 2.5 cm sampling area), manually homogenized, and stored at 4°C until processing, which was initiated within 48 h of sample collection. For rhizosphere samples, plants were excavated and roots with adhering soil were collected, homogenized, and stored at 4°C until laboratory processing.

Soil and plant chemistry

Bulk soil moisture was defined by measuring soil gravimetric water content according to standard oven-drying methods [1]. Briefly, fresh soil weights were recorded, samples were dried at an oven at 105°C until a constant weight was reached, and the dry soil weight was recorded to calculate the mass of water lost per unit mass of dry soil. Soil physicochemical properties for bulk soil samples at all timepoints were measured by Ward Laboratories (Kearney, NE, USA). Measured variables included soil pH, buffer pH, soluble salts, organic matter, nitrate, phosphorus, potassium, sulfate, and a suite of exchangeable cations and micronutrients using standard methods (Table S1).

Soil pH and soluble salts were measured using a 1:1 mixture, by weight, of 1 part soil to 1 part distilled H_2O [2]. Soil organic matter was determined as percent mass loss on ignition after heating sample in a muffle furnace to 500°C [3]. Nitrate and Ammonium were measured by colorimetry on 2M KCl extracts [4]. Potassium, calcium, magnesium, and sodium were quantified using ammonium acetate extractions [5]. Zinc, iron, manganese, and copper were measured using a diethylenetriaminepentaacetic acid (DTPA) extraction [6]. Phosphorus was measured using Olsen bi-carbonate solution extraction [7], and sulphite (sulfur trioxide) was measured using a Mehlich-3 extraction [8]. Cation exchange capacity (CEC) and percent base saturation values were calculated from the sum of extracted cations.

Plant growth, root AMF colonization and fruit quality parameters were assessed at different time points throughout the growing season. Root length colonized by arbuscular mycorrhizal fungi was quantified using a gridline intersect method [9] following root staining [10]. Briefly, roots were rinsed in PBS after rhizosphere soil collection, subsampled (0.1 g) and stored at 4°C. Root subsamples were then cut into approximately 2-centimeter segments and stained using a method modified from Vierheilig et al. (1998). Root segments were cleared in a 10% KOH solution at 90°C for 40 minutes and stained in a 5% ink and vinegar solution for 5 minutes at 90°C. Roots were then bleached in 10% sodium hypochlorite solution for 5 minutes. Roots were stained in 5% ink-vinegar solution and stored in 10% water solution prior to quantification. AMF colonization was visually quantified using the gridline intersect method (Giovannetti and Mosse, 1980) on a dissecting scope at x 80 magnification. A minimum of 100 root segments were counted for each sample.

Vine biomass and fruit yield estimates were obtained through destructive sampling. Dry vine biomass (kg/ha) was determined by harvesting vines from subsampled plants, drying at 60°C for four weeks, and extrapolating to plot-level area. Red, green, and total fruit hand harvest estimates (MT/ha) were calculated by collecting fruit from six randomly selected plants per plot, and extrapolating yields to the entire row based on the proportion of sampled distance to total row length. Total fruit yield was calculated as the sum of red and green fruit. A subsample of red fruits was collected from each subplot and taken to a Processing Tomato Advisory Board (http://www.ptab.org) grading station to measure total soluble solids, fruit color and fruit pH. Total soluble solids (°Brix) were measured using a refractometer on collected fruit samples. Fruit pH was determined on blended fruit samples as described above. Fruit color was quantified

as Hunter hue angle using a Konica Minolta model CR-410 colorimeter on whole tomatoes blended under high vacuum to remove air bubbles.

Plant tissue composed of three leaflets per plants mixed to form a composite sample representative of each plot were analyzed for macro- and micronutrient concentrations at Pennsylvania State University for nutrient analyses (State College, PA). Total nitrogen content (%) was determined by thermal conductivity using combustion analysis with an Elementar Vario Max N/C Analyzer, where dried plant material ignited in an induction furnace at approximately 900°C in a helium and oxygen environment within quartz tube [11]. Phosphorus, potassium, calcium, magnesium, and sulfur (%) along with manganese, iron, copper, boron, aluminum, zinc, and sodium (mg/kg) were determined following acid digestion [12]. Dried, ground plant material was mixed with an internal standard and pre-digestant, heated to 60°C in an aluminum block, digested with acid at 120°C, then diluted, filtered, and analyzed using ICP emission spectroscopy.

Virome DNA extraction, library construction, and shotgun sequencing

Samples were processed within 24 hours after collection for viromics and kept at 4°C until processing. For each bulk soil sample, 10 grams of soil were suspended in 30 mL of protein-supplemented phosphate-buffered saline solution (PPBS: 2% bovine serum albumin, 10% phosphate-buffered saline, 1% potassium citrate, and 150 mM $MgSO_4$) and then briefly vortexed and placed on an orbital shaker (30 min, 400 rpm, 4°C). For each rhizosphere sample, the roots were vigorously shaken to remove loose soil particles, and only the adhered portion was analyzed. The roots were suspended and shaken in the same PPBS buffer as for bulk soils, with no explicit differentiation between the rhizosphere and endosphere (i.e., the cut ends of roots were also exposed to the buffer). From this point, bulk soil and rhizosphere virome processing was the same. The shaken soil-buffer solution was centrifuged (10 min, 3,095 x g, 4°C) and the resulting supernatant was then centrifuged twice (8 min, 10,000 x g, 4°C) to remove residual soil particles. The centrifuged supernatants were then filtered through a 0.22 μm polyethersulfone membrane filter to remove most cells. The filtrate was then ultracentrifuged (2 hrs 25 min, 32,000 x rpm, 4°C) to pellet the virions, using an Optima LE-80K ultracentrifuge with a 50.2 Ti rotor (Beckman-Coulter Life Sciences). Supernatant was discarded, and pellets were resuspended in 100 μl of ultrapure water and treated with 10 units of RQ1 RNase-free DNase and 10 μl of

10× DNase buffer (Promega Corp., Madison, WI, USA) to remove free DNA. Samples were incubated at 37°C for 30 min, and the reaction was stopped by adding 10 µl of the DNase stop solution (Promega Corp., Madison, WI, USA). DNA was then extracted from the viral fraction, using the DNeasy PowerSoil Pro kit (Qiagen, Hilden, Germany), following the manufacturer's instructions, with an added step of a 10-minute incubation at 65 °C before the bead-beating step. Libraries were constructed using the DNA Hyper Prep library kit (Kapa Biosystems-Roche, Basel, Switzerland). Paired-end 150 bp sequencing was done using the NovaSeq S4 System (Illumina), to an approximate depth of 10 Gbp per virome.

Total DNA extraction, amplicon library construction, and sequencing

For each bulk soil sample, total DNA was extracted from 0.25 g of soil with the DNeasy PowerSoil Pro kit (Qiagen, Hilden, Germany), following the manufacturer's instructions, with an added step of a 10-minute incubation at 65°C prior to the bead-beating step. For each rhizosphere sample, 0.25 g of soil was brushed off the roots into the extraction tube, followed by the same extraction protocol. Construction of the amplicon libraries followed a previously described dual-indexing strategy [13, 14]. To target the V4 region of the 16S rRNA gene, universal primers 515F and 806R were used, using the following PCR protocol: an initial denaturation step at 98°C for 2 min, followed by 30 cycles of 98°C for 20 s, 50°C for 30 s and 72°C for 45 s, and a final extension step at 72°C for 10 min. To amplify the ITS1 region, we used the universal primers ITS1-F and ITS2 [15–17] and the following PCR program: an initial denaturation step at 95°C for 2 min, followed by 35 cycles of 95°C for 20 s, 50°C for 30 s, and 72°C for 50 s, followed by a final extension at 72°C for 10 min. All PCR reactions were performed using the Platinum Hot Start PCR Master Mix (Invitrogen). Libraries were cleaned using AmpureXP magnetic beads (Beckman-Coulter Life Sciences), quantified (Qubit 4 fluorometer, ThermoFisher Scientific), and pooled in equimolar concentrations. Paired-end sequencing (250 bp) was performed on the MiSeq System (Illumina), using a standard flow cell per library (one for 16S rRNA gene amplicons and one for ITS amplicons).

RNA extraction, library construction, and sequencing

Plants were excavated from the soil and vigorously shaken. Roots with adhering soil were collected as rhizosphere samples. Each rhizosphere sample was divided into three portions: one portion was kept at 4°C (on ice) for DNA extraction (as described above), another was flash-frozen in liquid nitrogen for RNA extraction, and a third portion was reserved for arbuscular mycorrhizal fungal (AMF) colonization assessment via root staining. Rhizosphere samples designated for RNA extraction were immediately flash-frozen in liquid nitrogen in the field and stored at -80°C until further processing. Total RNA was extracted using the RNeasy PowerSoil Pro Kit (Qiagen, Hilden, Germany), following the manufacturer's instructions. RNA was submitted to Genewiz (San Francisco, CA, USA) for ribodepletion, cDNA preparation, library construction via the NEBNext Ultra II RNA library prep kit (New England Biolabs, Ipswitch, MA, USA), and sequencing. Paired-end sequencing (150 bp) was done using the NovaSeq 6000 System (Illumina) to an approximate sequencing depth of 10 Gbp per sample.

DNA virome bioinformatic processing

Virome sequencing reads were trimmed using Trimmomatic v0.39 [18], removing Illumina adapters and quality trimming reads, using paired-end trimming, a sliding window size of 3:40, and a minimum read length of 50 bp. PhiX sequences were removed using bbduk from the BBMap v38.72 package [19], using k=31 and hdist=1, and host plant reads were removed using Bowtie2 v2.4.2 [20], using the sensitive setting, mapping against the genome of tomato (*S. lycopersicum*), GenBank accession number GCA_012431665. The remaining reads were assembled into contigs, using MEGAHIT v1.0.6 [21], with settings k-min 27, minimum contig length of 10 kbp, and presets meta-large. Resulting contigs were renamed using the rename function from BBMap, and viral contigs were predicted using VIBRANT v1.2.0 [22], in virome mode. All predicted viral contigs were dereplicated into vOTUs, using dRep v3.2.0 [23], at 95% average nucleotide identity (ANI) with a minimum alignment of 85%, using the ANImf algorithm. Reads were mapped to the vOTUs and to the PIGEONv2.0 database [24] using Bowtie2 v2.4.2 [20], using sensitive mode. vOTUs from the PIGEONv2.0 database that were recovered in this dataset were clustered with the vOTUs assembled from this dataset using dRep, using the same settings as above. Reads were then again mapped to this non-redundant dataset of vOTUs, using Bowtie2, and the resulting samfiles were converted to bamfiles using SAMtools

v1.15.1 [25]. The resulting non-redundant dataset of vOTUs was taxonomic annotated using v1.11.1 [26]. A coverage table was created with CoverM v0.6.1 [27] using CoverM contig with the mean coverage and a minimum covered fraction (breadth) of 75%. The resulting coverage table was used for statistical analysis, unless otherwise noted. All scripts for bioinformatic processing are available on Github (https://github.com/AnneliektH/TomatoRhizo).

Amplicon sequence bioinformatic processing

Paired-end reads were assembled into single sequences using PANDAseq v2.9 [28]. Chimeric sequences were removed, reads were dereplicated, and denoising and read merging were performed using DADA2 v1.12.1 with default settings, following the standard DADA2 workflow ([29]; https://benjjneb.github.io/dada2/tutorial.html). Taxonomy was assigned using the RDP classifier implementation in DADA2 [30] with the SILVA database v132 [31] for 16S rRNA gene sequences, and with the UNITE database v2021-05-10 for ITS sequences [32]. Amplicon sequence variant (ASV) tables with raw read counts per sample were generated using the `makeSequenceTable` function in DADA2. Sequences matching mitochondria or chloroplasts (0.11% of total 16S rRNA gene reads) were removed from the 16S rRNA gene dataset using phyloseq v1.52.0 [33]. ASV tables were converted to relative abundances prior to downstream analyses.

Metatranscriptome bioinformatic processing for both RNA viral community and community transcript analyses

Paired-end metatranscriptomic reads were first trimmed, and PhiX and the host plant (tomato) were removed, following the same procedures used for DNA viromes above. To optimize the recovery of low-abundance transcripts, the remaining host-filtered reads were co-assembled using MEGAHIT v1.0.6 [21] by grouping individual samples according to their plot location and treatment condition, with settings k-min 27, minimum contig length of 200 bp, and the meta-large preset. Predicted proteins were generated from assembled contigs using Prodigal v2.6.3 [34], using standard settings. For RNA viral analyses, HMMER v3.3.2 [35] was used to recover RNA-dependent RNA polymerase (RdRp) sequences with a E-value threshold of 1e-05,

as previously described [36, 37]. Predicted proteins were further annotated using DIAMOND v0.9.22.123 [38] against the NCBI nr prokaryotic database (v203), using a E-value threshold of 1e-06. Contigs containing prokaryotic genes were flagged and reads mapping to these contigs were removed to reduce prokaryotic RNA signal. The remaining reads were re-assembled using MEGAHIT, and this process was iterated three times. The final set of contigs containing predicted RdRp sequences was clustered using dRep v3.2.0 [23] at 95% ANI with a minimum alignment of 85%, using the ANImf algorithm. Metatranscriptomic reads were mapped back to these sequences and to RefSeq v203 RNA viral sequences (n = 4,472) using Bowtie2, and a coverage table was generated using CoverM v0.6.1 [27] with the same parameters as described for DNA viromes. For community transcriptome analyses, reads remaining after host (tomato) removal were further processed to reduce rRNA contamination. rRNA reads were removed using SortMeRNA v4.3.7 [39] with default rRNA reference databases (v4.3.4), and unaligned reads were retained for downstream analyses. Because SortMeRNA outputs interleaved reads, BBTools repair.sh [19] was used to restore separate forward and reverse read files. Contigs generated from the plot location and treatment condition co-assemblies described above were concatenated into a single unified reference transcriptome database and this was used to map the non-rRNA reads back to. Salmon v1.10.3 index [40] of these pooled contigs was built in quasi-mapping mode, and transcript abundance was quantified per sample using Salmon with automatic library type detection and default parameters. Salmon outputs transcript-level read estimates that account for sequence composition and multi-mapping reads [40]. Quantification outputs from all samples were merged using Salmon quantmerge to generate a matrix of raw read counts for downstream analyses. For functional annotation, open reading frames (ORFs) were predicted from contigs using TransDecoder v5.7.1 [41], and long ORFs were translated into protein sequences. Proteins were annotated using eggNOG-mapper v2.1.13 [42]. Contig-level functional annotations were linked to transcript abundance for downstream analyses. Community metatranscriptome data processing scripts are available on Github (https://github.com/ljiraska/Tomato_rhizo_AMF).

Statistical analysis

All statistical analyses were performed using R v4.5.2 [43], using the mean coverage vOTU abundance table or the other coverage tables prepared as described above (for RdRps, transcript table, 16S rRNA gene ASVs, and ITS ASVs, respectively), and ggplot2 v4.0.3 package [44], unless otherwise noted. Phyloseq objects were prepared with abundance tables and metadata using R package phyloseq v1.52.0 [33]. ASV tables were CSS normalized and all tables were Hellinger transformed with transform function from microbiome v1.30.0 [45] package. Statistical significance was defined as $p < 0.05$ for single comparisons and adjusted $p < 0.05$ (Benjamini-Hochberg correction) for multiple comparisons unless otherwise noted.

Alpha-diversity metrics (observed richness) were calculated using microbiome v1.30.0 [45] using function alpha. Statistical significance between bulk and rhizosphere for observed richness, and DNA concentration was assessed using rstatix v0.7.2 [46] using wilcox_test function for a Wilcoxon rank-sum test and for plant related variables using t_test function for t-test.

Principal coordinate analysis (PCoA) with Bray-Curtis dissimilarity matrices were calculated for viral, bacterial and fungal communities using the ordinate function from the phyloseq package v1.52.0 [33]. Canonical analysis of principal coordinates (CAP) was conducted to assess the effect of plot on community structure using Bray-Curtis distances with the formula ~Plot, also with ordinate function. Soil chemistry and plant chemistry data were each standardized (mean-centered and scaled to unit variance) prior to PCA using the scale function in base R. Moisture content was excluded from soil chemistry analysis for PCA and PCA was performed with prcomp function in base R.

Permutational multivariate analysis of variance (PERMANOVA) was conducted using the adonis2 function from the vegan package v2.7.2 (Oksanen et al., 2022) to test the effects of sample type (bulk vs. rhizosphere), treatment, timepoint, and plot on community composition based on Bray-Curtis dissimilarity matrices and using scaled soil chemistry and plant data with Euclidean distance matrices. PERMANOVA was performed with 999 permutations, and effects were evaluated sequentially by terms. Where appropriate, two- and three-way interactions were also tested. Mantel tests were performed to assess correlations between community (using Bray-Curtis) and soil chemistry (Euclidean) dissimilarity matrices of different organisms using the mantel function from the vegan package with 999 permutations and Pearson correlation.

Linear regressions and Pearson's correlation tests were performed to assess relationships between PCoA axis 1 scores and environmental variables (e.g., soil moisture) using the cor.test function in base R.

Variation partitioning analysis was conducted following the approach described by Legendre and Legendre (2012) to decompose the variation in community composition explained by soil moisture, selected soil chemistry variables, location of sampling, and temporal factors. Forward selection of soil chemistry variables was performed using the forward.sel function from the adespatial v0.3.28 package, with the adjusted R^2 threshold set to the global adjusted R^2 of the full soil chemistry model and 999 permutations. When forward selection identified significant variables, only these selected variables were retained for soil chemistry in the subsequent variation partitioning. Variation partitioning was performed using the varpart function from the vegan package. For temporal effects, variation partitioning was performed separately using categorical timepoint (four discrete sampling dates) and linear time (weeks since first sampling) to compare discrete versus continuous temporal models.

Heatmaps of scaled bulk soil chemistry data were generated using the pheatmap function from the pheatmap package v1.0.13 [47] with hierarchical clustering of samples based on Euclidean distance (default settings).

Differential gene expression analysis was performed using DESeq2 v1.48.2 [48], following the methods described in the tutorial of the package vignette (https://bioconductor.org/packages/devel/bioc/vignettes/DESeq2/inst/doc/DESeq2.html#principal-component-plot-of-the-samples). A DESeqDataSet object was constructed with a design formula accounting for site, timepoint, and treatment effects (~ Site + Timepoint + Treatment). Differential expression testing was conducted using the default Wald test, comparing AMF treatment versus control samples. Log2 fold changes were shrunk using the apeglm method to reduce noise from low-count genes. Transcripts with adjusted p-value < 0.05 (Benjamini-Hochberg correction) and absolute log2 fold change > 1 were considered differentially expressed genes. Functional annotation of transcripts was obtained from eggNOG-mapper (as described above), with isoform suffixes removed and the best annotation per transcript retained based on lowest e-value. For community gene expression data, principal component analysis (PCA) was performed on variance-stabilized transformed count data using the plotPCA function from DESeq2 v1.48.2 [48]. The analysis included timepoint, treatment, and site as grouping variables.

To evaluate desiccation stress in rhizosphere microbial communities, we used 22 previously identified desiccation-upregulated genes in soil bacterium *Curtobacterium* [49]. We filtered metatranscriptomic data to genes including those involved in osmolyte synthesis (*dhaK, dhaM, treZ, treY, otsB),* DNA repair and recombination (*recA, recN, uvrA, dps*), SOS response (*lexA, recX*), oxidative stress response (*katA, ahpF*), efflux pumps (*macB, arsB, cnrH*), sporulation and stress sigma factors (*gdh, sigK, rpoE*), and other desiccation-related functions (*tgnD, gabD, fadA*) [49]. Relative abundance of desiccation genes was calculated as the sum of desiccation gene transcript abundance divided by total transcript abundance per sample. Temporal trends were analyzed using linear mixed-effects models with lme4 v1.1.37 package [50] with time (weeks) and treatment, as fixed effects and plot location as a random effect. P-values were obtained using the lmerTest v3.1.3 package [51]. Treatment-specific temporal slopes were calculated using the emmeans v1.11.2.8 package [52].

All maps were made using the R packages rnaturalearth v1.2.0 [53], rnaturalearthdata v1.0.0 [54] and sf v1.0.21 [55], and all other plots were created using the R package ggplot2 v4.0.0 [56], and eulerr v7.0.4 [57]. Plots were composed together with R package patchwork v1.3.2 [58] and Affinity Designer 2 was used to make modification to improve readability of figures. For data manipulation we used R packages tidyverse v2.0.0 [59], dplyr v1.1.4 [60] and stringr v1.5.2 [61]. All data processing and statistical analysis scripts are available on Github (https://github.com/ljiraska/Tomato_rhizo_AMF).

Supplementary figures

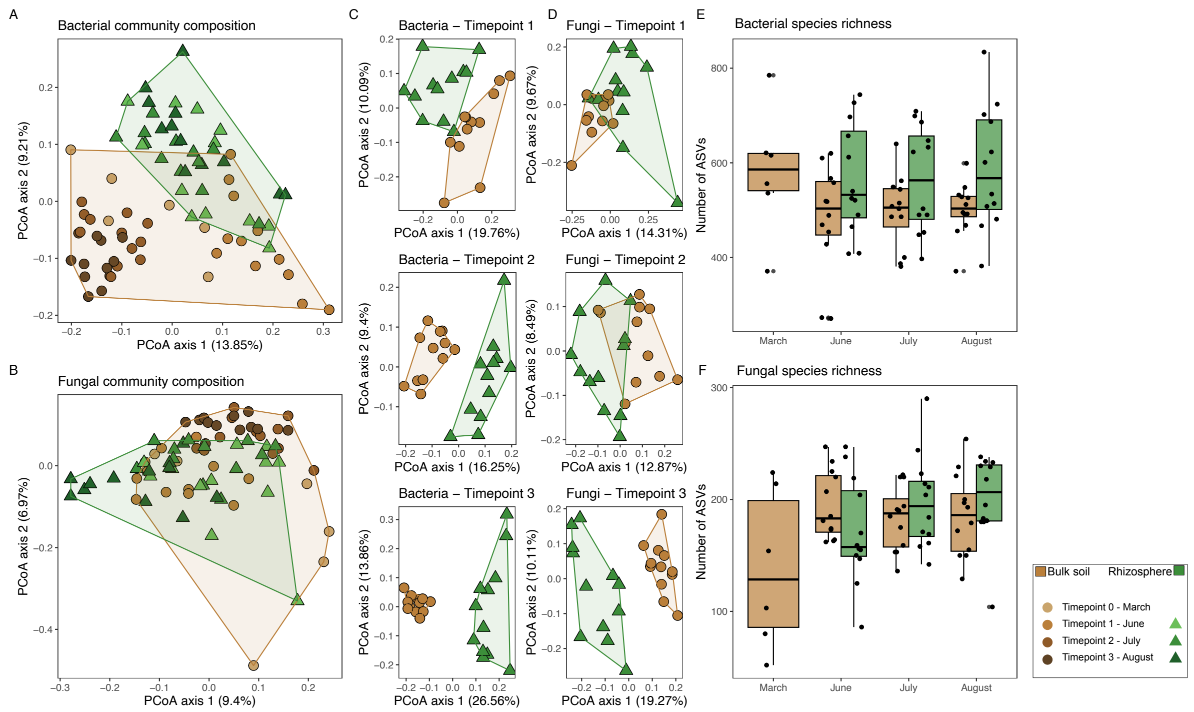

Figure S1. Soil bacterial and fungal community composition and ASV richness patterns. (A–B) Principal Coordinates Analysis (PCoA) based on Bray–Curtis dissimilarities calculated from amplicon sequence variant (ASV) abundance tables for bacterial (A) and fungal (B) communities. Points are colored by soil compartment and sampling time point. Sampling times were T0 = March (pre-planting), T1 = June (6 weeks after planting), T2 = July (10 weeks after planting), and T3 = August (17 weeks after planting). (C–D) PCoA plots of bacterial (C) and fungal (D) communities faceted by sampling time and colored by soil compartment. (E–F) Total numbers of bacterial (E) and fungal (F) ASVs (richness) recovered at each sampling time point in bulk and rhizosphere soils. Colors correspond to the legend in panel A. Boxes represent the interquartile range (IQR, 25th–75th percentile), the central line shows the median, whiskers extend to 1.5× IQR, and individual points outside whiskers are outliers. Points are jittered for clarity. Statistical significance between bulk and rhizosphere ASVs richness (E-F) was assessed using a Wilcoxon rank-sum test and is indicated where applicable. ns., not significant; *$p < 0.05$; **$p < 0.01$; ***$p < 0.001$; ****$p < 0.0001$.

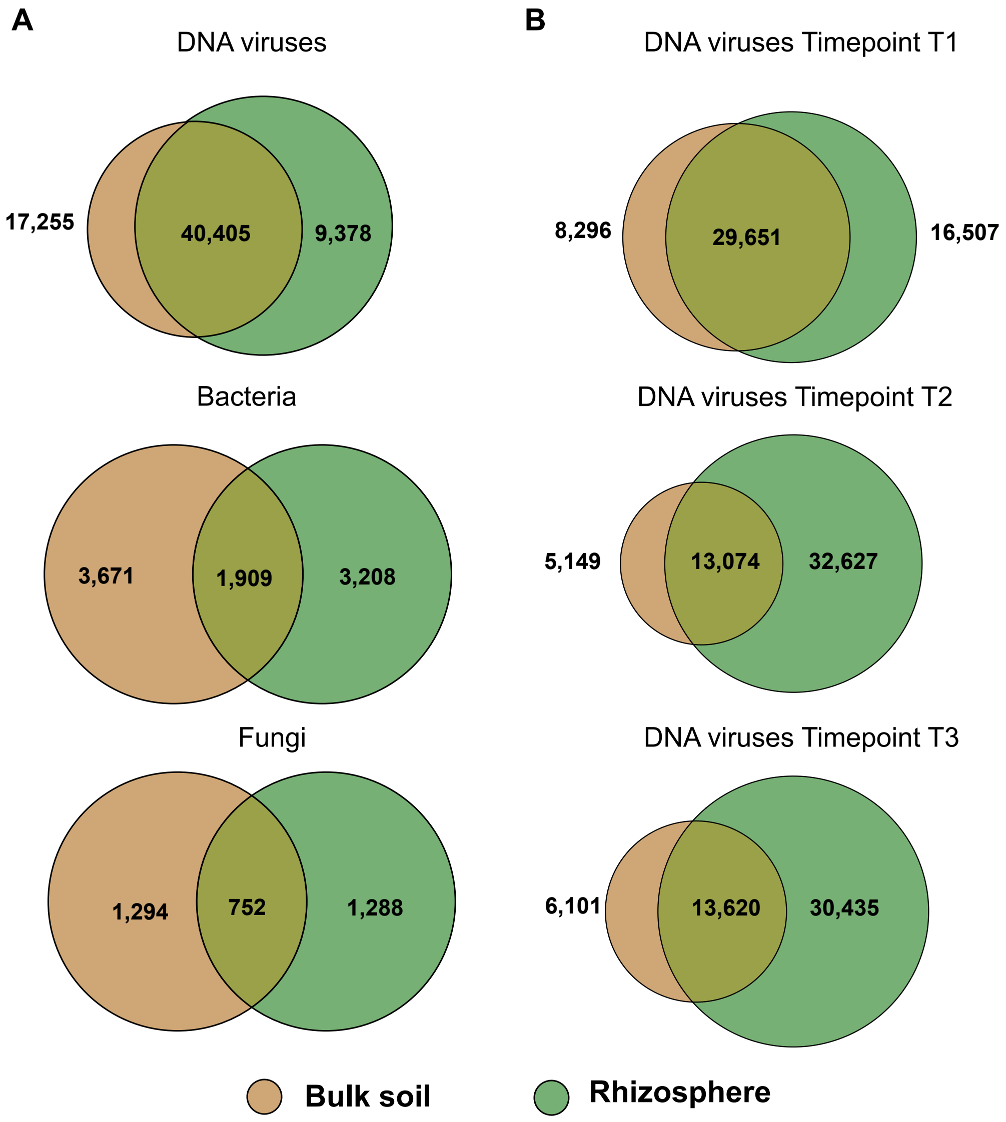

Figure S2. Euler diagrams showing detection overlap of vOTUs (DNA viruses) and ASVs (bacteria and fungi) across soil compartments and time. (A) Overlap between bulk soil and rhizosphere compartments for DNA viruses, bacteria, and fungi; numbers indicate shared and compartment-specific taxa. (B) Overlap of DNA viral vOTUs between bulk soil and rhizospheres across sampling time points (T1–T3). The colors correspond to different compartments.

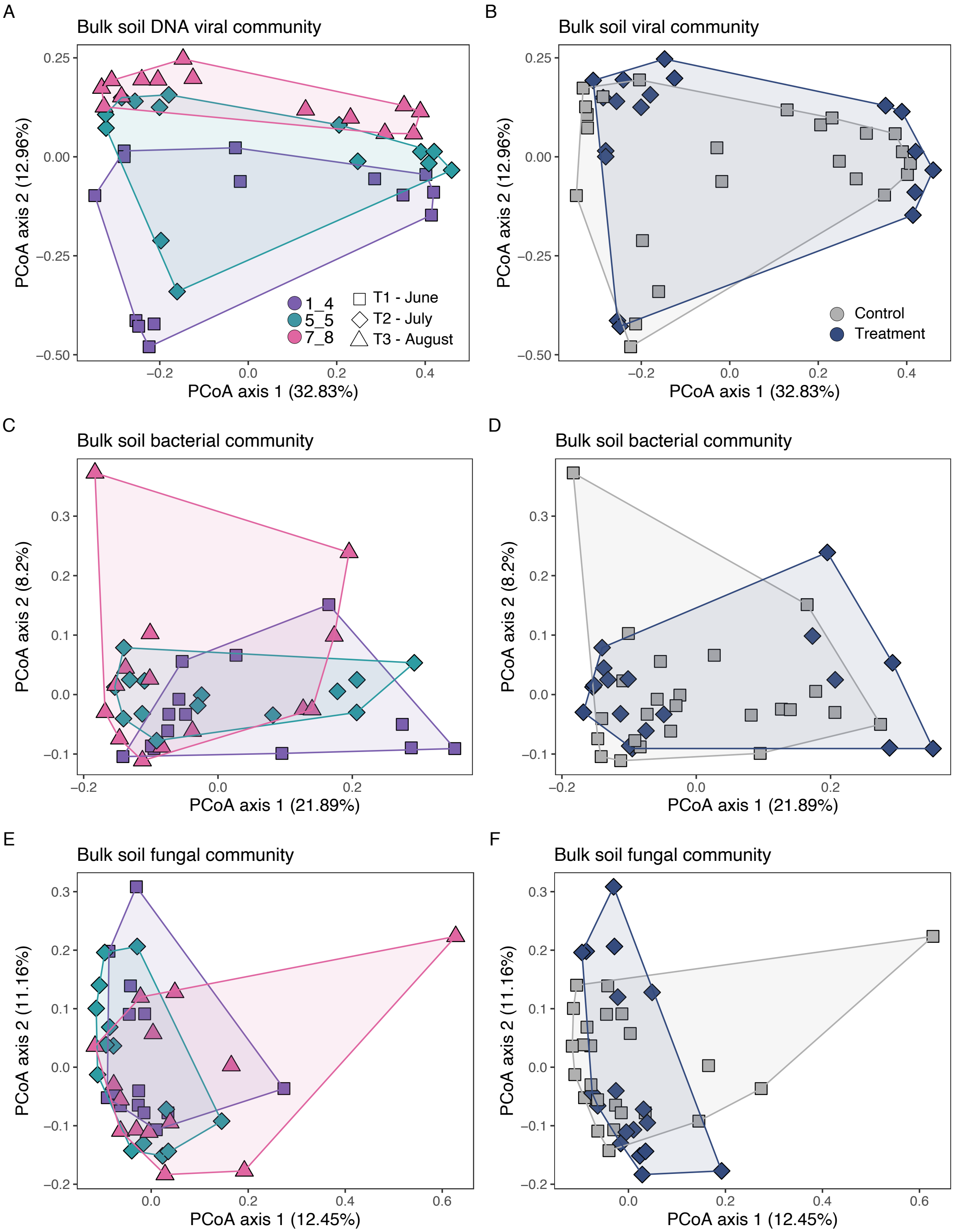

Figure S3. Principal coordinates analyses (PCoA) based on Bray–Curtis dissimilarities of bulk soil DNA viral, bacterial, and fungal community composition. (A–B) DNA viral communities,

with samples colored by plot location (A) and by AMF treatment versus control (B). (C–D) Bacterial communities, colored by plot location (C) and by AMF treatment versus control (D). (E–F) Fungal communities, colored by plot location (E) and by AMF treatment versus control (F).

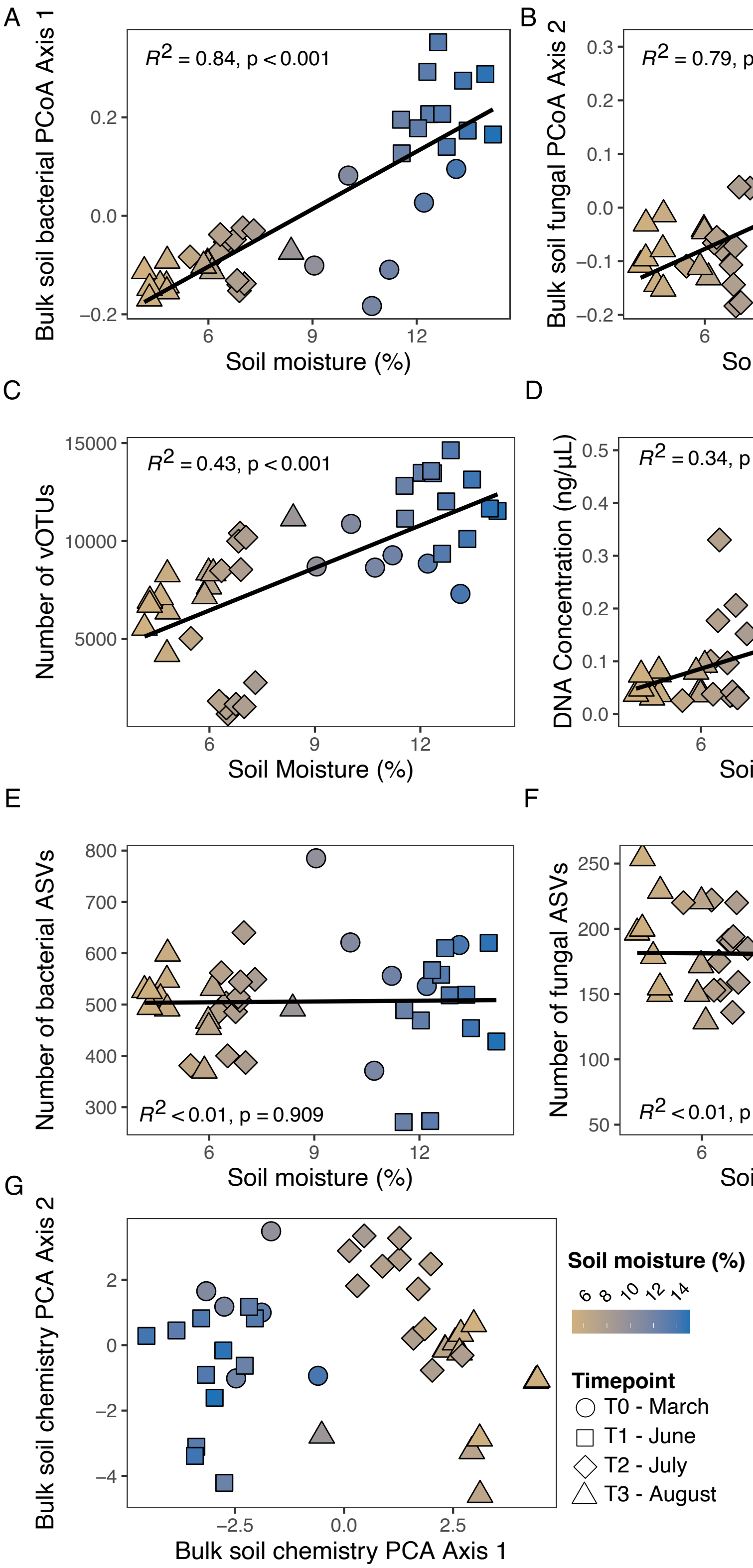

A
R^2 = 0.84, p < 0.001
Bulk soil bacterial PCoA Axis 1
Soil moisture (%)
B
R^2 = 0.79, p < 0.001
Bulk soil fungal PCoA Axis 2
Soil moisture (%)
C
R^2 = 0.43, p < 0.001
Number of vOTUs
Soil Moisture (%)
D
R^2 = 0.34, p < 0.001
DNA Concentration (ng/µL)
Soil Moisture (%)
E
R^2 < 0.01, p = 0.909
Number of bacterial ASVs
Soil moisture (%)
F
R^2 < 0.01, p = 0.923
Number of fungal ASVs
Soil moisture (%)
G
Bulk soil chemistry PCA Axis 2
Bulk soil chemistry PCA Axis 1
Soil moisture (%)
6 8 10 12 14
Timepoint
T0 - March
T1 - June
T2 - July
T3 - August

Figure S4. Pearson correlations between bulk soil moisture and microbial and viral community metrics in bulk soils. Scatter plots show relationships between soil moisture and (A) bacterial community composition (PCoA axis 1), (B) fungal community composition (PCoA axis 2), (C) DNA viral richness, (D) DNA concentration as a proxy for viral biomass, and bacterial (E) and fungal (F) richness. Points are colored by soil moisture content (values according to the x-axis of A), shapes are sampling time point.

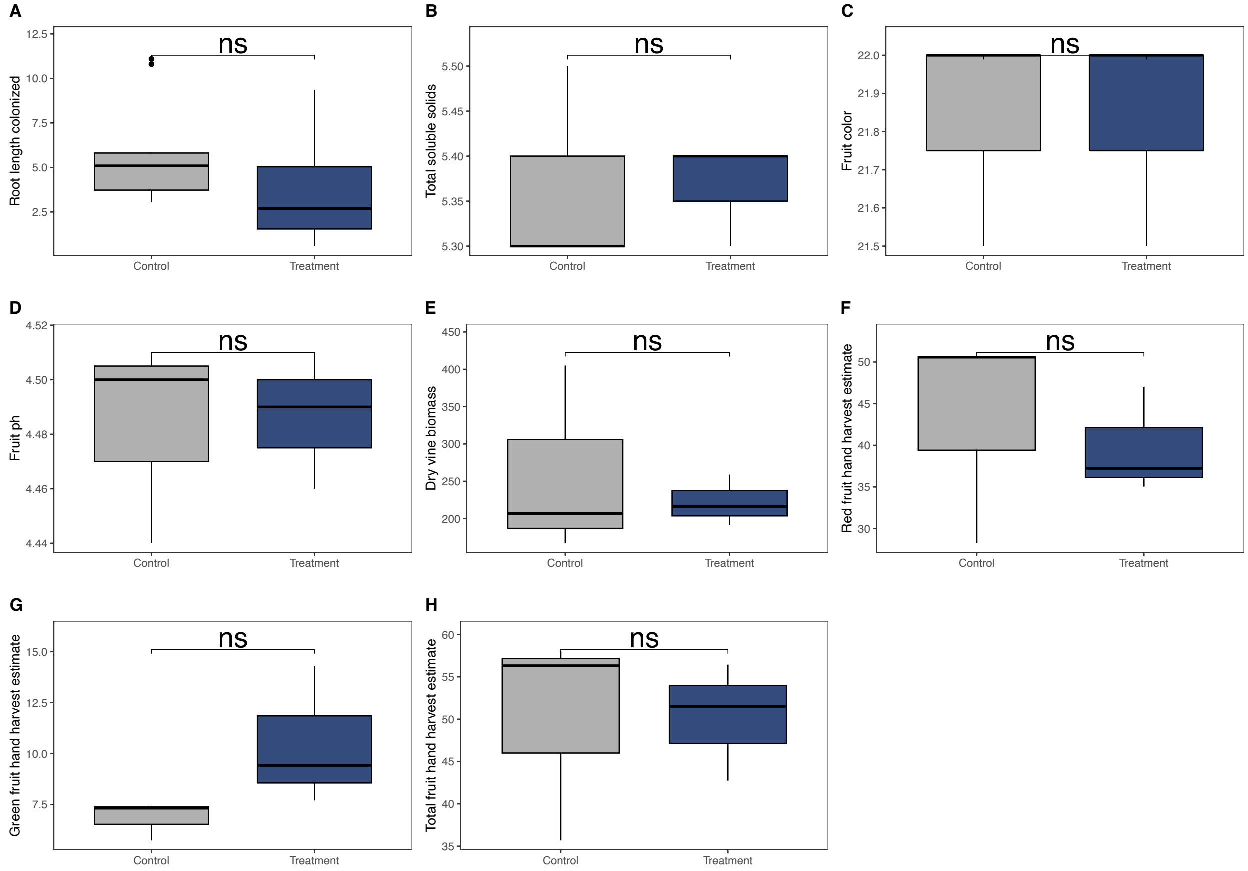

Figure S5. Lack of any measured effects of AMF treatment on plant performance and fruit traits. Boxplots compare control and AMF-treated plants for (A) root length colonized by AMF and (B-F) at harvest T3 timepoints only; (B) total soluble solids, (C) fruit color, (D) fruit pH, (E) dry vine biomass, (F) red fruit hand harvest estimate, (G) green fruit hand harvest estimate, and (H) total fruit hand harvest estimate. Statistical differences between treatments were assessed using Wilcoxon rank-sum tests; no variables showed significant differences between control and AMF treatments ($p > 0.05$ for all comparisons). Boxes represent the interquartile range (IQR, 25th–75th percentile), the central line shows the median, whiskers extend to 1.5× IQR, and individual points outside whiskers are outliers. $p > 0.05$ for all comparisons; ns., not significant

References:

1. Black CA et al. Methods of Soil Analysis (Part 1). Physical and Mineralogical Properties Including Statistics of Measurements and Sampling. Madison: American Society of Agronomy Inc., 1965.
2. Eckert DJ. Recommended pH and lime requirement tests. *Recomm Chem Soil Test Proced North Cent Reg North Cent Reg Publ* 1988;**221**:6–8.
3. Heiri O, Lotter AF, Lemcke G. Loss on ignition as a method for estimating organic and carbonate content in sediments: reproducibility and comparability of results. *J Paleolimnol* 2001;**25**:101–110.
4. Keeney DR, Nelson DW. Nitrogen—inorganic forms. *Methods Soil Anal Part 2 Chem Microbiol Prop* 1982;**9**:643–698.
5. Normandin V, Kotuby-Amacher J, Miller RO. Modification of the ammonium acetate extractant for the determination of exchangeable cations in calcareous soils. *Commun Soil Sci Plant Anal* 1998;**29**:1785–1791.
6. Liang J, Karamanos RE. DTPA-extractable Fe, Mn, Cu and Zn. *Soil Sampl Methods Anal* 1993;87–90.
7. Sims JT. Soil test phosphorus: Olsen P. *Methods Phosphorus Anal Soils Sediments Residuals Waters* 2000;**20**.
8. Mehlich A. Mehlich 3 soil test extractant: A modification of Mehlich 2 extractant. *Commun Soil Sci Plant Anal* 1984;**15**:1409–1416.
9. Giovannetti M, Mosse B. An evaluation of techniques for measuring vesicular arbuscular mycorrhizal infection in roots. *New Phytol* 1980;489–500.

10. Vierheilig H et al. Ink and Vinegar, a Simple Staining Technique for Arbuscular-Mycorrhizal Fungi. *Appl Environ Microbiol* 1998;**64**:5004–5007. https://doi.org/10.1128/AEM.64.12.5004-5007.1998
11. Horneck DA, Miller RO. Determination of total nitrogen in plant tissue. *Handb. Ref. Methods Plant Anal.* 1998. CRC Press, 1998, pp 75–83.
12. Huang C-YL, Schulte EE. Digestion of plant tissue for analysis by ICP emission spectroscopy. *Commun Soil Sci Plant Anal* 1985;**16**:943–958.
13. Caporaso JG et al. QIIME allows analysis of high-throughput community sequencing data. *Nat Methods* 2010;**7**:335–336. https://doi.org/10.1038/nmeth.f.303
14. Edwards J, Santos-Medellín C, Sundaresan V. Extraction and 16S rRNA Sequence Analysis of Microbiomes Associated with Rice Roots. *Bio-Protoc* 2018;**8**:e2884. https://doi.org/10.21769/BioProtoc.2884
15. Agler MT et al. Microbial Hub Taxa Link Host and Abiotic Factors to Plant Microbiome Variation. *PLoS Biol* 2016;**14**:e1002352. https://doi.org/10.1371/journal.pbio.1002352
16. Gardes M, Bruns TD. ITS primers with enhanced specificity for basidiomycetes - application to the identification of mycorrhizae and rusts. *Mol Ecol* 1993;**2**:113–118. https://doi.org/10.1111/j.1365-294X.1993.tb00005.x
17. White TJ et al. Amplification and direct sequencing of fungal ribosomal RNA genes for phylogenetics. *PCR Protoc Guide Methods Appl* 1990;**18**:315–322.
18. Bolger AM, Lohse M, Usadel B. Trimmomatic: a flexible trimmer for Illumina sequence data. *Bioinformatics* 2014;**30**:2114–2120. https://doi.org/10.1093/bioinformatics/btu170
19. Bushnell B. BBMap: A Fast, Accurate, Splice-Aware Aligner. Lawrence Berkeley National Lab. (LBNL), Berkeley, CA (United States), 2014.

20. Langmead B, Salzberg SL. Fast gapped-read alignment with Bowtie 2. *Nat Methods* 2012;**9**:357–359. https://doi.org/10.1038/nmeth.1923

21. Li D et al. MEGAHIT: an ultra-fast single-node solution for large and complex metagenomics assembly via succinct de Bruijn graph. *Bioinforma Oxf Engl* 2015;**31**:1674–1676. https://doi.org/10.1093/bioinformatics/btv033

22. Kieft K, Zhou Z, Anantharaman K. VIBRANT: automated recovery, annotation and curation of microbial viruses, and evaluation of viral community function from genomic sequences. *Microbiome* 2020;**8**:90. https://doi.org/10.1186/s40168-020-00867-0

23. Olm MR et al. dRep: a tool for fast and accurate genomic comparisons that enables improved genome recovery from metagenomes through de-replication. *ISME J* 2017;**11**:2864–2868. https://doi.org/10.1038/ismej.2017.126

24. ter Horst AM et al. Dispersal, habitat filtering, and eco-evolutionary dynamics as drivers of local and global wetland viral biogeography. *ISME J* 2023;**17**:2079–2089. https://doi.org/10.1038/s41396-023-01516-8

25. Li H et al. The Sequence Alignment/Map format and SAMtools. *Bioinforma Oxf Engl* 2009;**25**:2078–2079. https://doi.org/10.1093/bioinformatics/btp352

26. Camargo AP et al. Identification of mobile genetic elements with geNomad. *Nat Biotechnol* 2023;1–10. https://doi.org/10.1038/s41587-023-01953-y

27. Aroney STN et al. CoverM: read alignment statistics for metagenomics. *Bioinformatics* 2025;**41**:btaf147. https://doi.org/10.1093/bioinformatics/btaf147

28. Masella AP et al. PANDAseq: paired-end assembler for illumina sequences. *BMC Bioinformatics* 2012;**13**:31. https://doi.org/10.1186/1471-2105-13-31

29. Callahan BJ et al. DADA2: High-resolution sample inference from Illumina amplicon data. *Nat Methods* 2016;**13**:581–583. https://doi.org/10.1038/nmeth.3869

30. Wang Q et al. Naive Bayesian classifier for rapid assignment of rRNA sequences into the new bacterial taxonomy. *Appl Environ Microbiol* 2007;**73**:5261–5267. https://doi.org/10.1128/AEM.00062-07

31. Quast C et al. The SILVA ribosomal RNA gene database project: improved data processing and web-based tools. *Nucleic Acids Res* 2013;**41**:D590-596. https://doi.org/10.1093/nar/gks1219

32. Abarenkov K et al. UNITE QIIME release for eukaryotes 2. 2020. 2020.

33. McMurdie PJ, Holmes S. phyloseq: An R Package for Reproducible Interactive Analysis and Graphics of Microbiome Census Data. *PLOS ONE* 2013;**8**:e61217. https://doi.org/10.1371/journal.pone.0061217

34. Hyatt D et al. Prodigal: prokaryotic gene recognition and translation initiation site identification. *BMC Bioinformatics* 2010;**11**:119. https://doi.org/10.1186/1471-2105-11-119

35. Eddy SR. Accelerated Profile HMM Searches. *PLOS Comput Biol* 2011;**7**:e1002195. https://doi.org/10.1371/journal.pcbi.1002195

36. Starr EP et al. Metatranscriptomic reconstruction reveals RNA viruses with the potential to shape carbon cycling in soil. *Proc Natl Acad Sci* 2019;**116**:25900–25908. https://doi.org/10.1073/pnas.1908291116

37. ter Horst AM et al. RNA Viral Communities Are Structured by Host Plant Phylogeny in Oak and Conifer Leaves. *Phytobiomes J* 2023;**7**:288–296. https://doi.org/10.1094/PBIOMES-12-21-0080-R

38. Buchfink B, Xie C, Huson DH. Fast and sensitive protein alignment using DIAMOND. *Nat Methods* 2015;**12**:59–60. https://doi.org/10.1038/nmeth.3176

39. Kopylova E, Noé L, Touzet H. SortMeRNA: fast and accurate filtering of ribosomal RNAs in metatranscriptomic data. *Bioinformatics* 2012;**28**:3211–3217. https://doi.org/10.1093/bioinformatics/bts611

40. Patro R et al. Salmon provides fast and bias-aware quantification of transcript expression. *Nat Methods* 2017;**14**:417–419. https://doi.org/10.1038/nmeth.4197

41. Haas BJ. TransDecoder. 2024. 2024.

42. Cantalapiedra CP et al. eggNOG-mapper v2: Functional Annotation, Orthology Assignments, and Domain Prediction at the Metagenomic Scale. *Mol Biol Evol* 2021;**38**:5825–5829. https://doi.org/10.1093/molbev/msab293

43. R Core Team. R: A Language and Environment for Statistical Computing. 2025. Vienna, Austria: R Foundation for Statistical Computing, 2025.

44. Wickham H et al. ggplot2: Create Elegant Data Visualisations Using the Grammar of Graphics. 2024. 2024.

45. Lahti L, Shetty S. microbiome: Microbiome Analytics. 2022. Bioconductor version: Release (3.15), 2022.

46. Kassambara A. rstatix: Pipe-Friendly Framework for Basic Statistical Tests. 2025. 2025.

47. Kolde R. pheatmap: Pretty Heatmaps. 2025. 2025.

48. Love M et al. DESeq2: Differential gene expression analysis based on the negative binomial distribution. 2023. Bioconductor version: Release (3.16), 2023.

49. Scales NC et al. Desiccation induces varied responses within a soil bacterial genus. *Environ Microbiol* 2023;**25**:3075–3086. https://doi.org/10.1111/1462-2920.16494

50. Bates D et al. lme4: Linear Mixed-Effects Models using ‘Eigen’ and S4. 2022. 2022.
51. Kuznetsova A et al. lmerTest: Tests in Linear Mixed Effects Models. 2026. 2026.
52. Lenth RV et al. emmeans: Estimated Marginal Means, aka Least-Squares Means. 2025. 2025.
53. Massicotte P, South A, Hufkens K. rnaturalearth: World Map Data from Natural Earth. 2026. 2026.
54. South A, Michael S, Massicotte P. rnaturalearthdata: World Vector Map Data from Natural Earth Used in ‘rnaturalearth’. 2024. 2024.
55. Pebesma E et al. sf: Simple Features for R. 2026. 2026.
56. Wickham H et al. ggplot2: Create Elegant Data Visualisations Using the Grammar of Graphics. 2026. 2026.
57. Larsson J et al. eulerr: Area-Proportional Euler and Venn Diagrams with Ellipses. 2025. 2025.
58. Pedersen TL. patchwork: The Composer of Plots. 2025. 2025.
59. Wickham H, RStudio. tidyverse: Easily Install and Load the ‘Tidyverse’. 2023. 2023.
60. Wickham H et al. dplyr: A Grammar of Data Manipulation. 2026. 2026.
61. Wickham H, Software P, PBC. stringr: Simple, Consistent Wrappers for Common String Operations. 2025. 2025.
